# Sparse Linear Algebra Accelerates Genotype Representation Graph Computation at Biobank Scale

**DOI:** 10.64898/2026.09.10.750583

**Published:** 2026-09-21

**Authors:** Yifan Li, Qingyao Sun, Drew DeHaas, Max Xiaohang Zhao, Adam R. Boyko, Shaila A. Musharoff, Xinzhu Wei, Giulia Guidi

**Affiliations:** 1 Department of Computer Science, Cornell University, 107 Hoy Rd, Ithaca, 14853, New York, USA.; 2 School of Operations Research and Information Engineering, Cornell University, 136 Hoy Rd, Ithaca, 14853, New York, USA.; 3 Department of Computational Biology, Cornell University, 350 Tower Rd, Ithaca, 14850, New York, USA.; 4 Cerebras Systems Inc, 1237 E. Arques Avenue, Sunnyvale, 94085, California, USA.; 5 Department of Biomedical and Translational Sciences, Cornell University, 350 Tower Rd, Ithaca, 14850, New York, USA.; 6 Applied Mathematics and Computational Research Division, Lawrence Berkeley National Laboratory, 1 Cyclotron Road, Berkeley, 94720, California, USA.

**Keywords:** Biobank-scale genomics, Genotype representation graph, Sparse linear algebra, GPU acceleration, Genome-wide association studies

## Abstract

Biobank-scale genomic analyses are increasingly constrained by computational costs, as hundreds of thousands to millions of samples and variants must be analyzed together. The genotype representation graph (GRG) compactly encodes population genetic variation to accelerate computation, but the current approach does not exploit modern accelerator architectures. This work introduces Mikado, a new methodology for expressing GRG-based computation, previously performed by graph traversal, using sparse linear algebra primitives. Under a reverse topological ordering of the graph nodes, the GRG adjacency matrix is strictly block-lower-triangular, and the genotype matrix-vector product becomes a sparse triangular solve that can be further decomposed into a pipelined sequence of blocked sparse matrix-vector multiplies. By decoupling computation from graph representation, our approach exposes fine-grained parallelism and enables hardware-optimized sparse primitives on GPUs. Mikado achieves an order-of-magnitude speedup and cost savings for PCA and BOLT-LMM compared with the original GRG traversal approach, including on *All of Us* cohorts. It provides a scalable, hardware-portable, researcher-friendly tool for population genetics at biobank scale.

## 1 Introduction

Over the past decade, the scale of population genomics data has grown dramatically. Biobank initiatives such as the *All of Us* Research Program now include hundreds of thousands to a million phased whole genomes [1], along with environmental and phenotypic data. This growth improves statistical power for genome-wide association studies (GWAS), heritability estimation, fine mapping, and emerging applications such as gene–environment interaction (G×E) testing [2–7]. The computational demands of these analyses, however, are rapidly outpacing conventional approaches. Common tabular formats such as VCF, BED (PLINK1), and BGEN represent each variant independently and do not exploit the shared relatedness structure underlying population genetic variation [2, 3]. PGEN (PLINK2) can optionally exploit linkage disequilibrium (LD) among nearby single nucleotide polymorphisms (SNPs) to reduce storage [8], but leaves redundancy in downstream statistical computation unaddressed. In benchmarks reported in concurrent work [9], principal component analysis (PCA) on simulated cohorts of 100k to 1 million individuals (14.5–23.7 million variants) in PGEN format took from several hours to more than 150 hours on a server with two AMD EPYC 7532 32-core processors and 1 TiB RAM. Tabular formats at biobank scale require substantial memory and compute, creating a bottleneck for downstream statistical analyses [6].

This work introduces Mikado^1^, a general sparse linear algebra formulation that makes operations on compressed genotype representations efficient and portable on modern accelerators. Its generality rests on a single primitive: repeated matrix–vector multiplication with the genotype matrix, which governs the scalability of many statistical genetics methods and, in some cases, also determines their accuracy.

In mixed-model association testing, such as BOLT-LMM [10], this product is the core operation in the inner loop of the conjugate gradient solver. In randomized Haseman–Elston regression [11], multiplying the genotype matrix by random vectors dominates the runtime, and faster trace estimation directly reduces the standard error of heritability estimates. The same product underlies randomized PCA, where the top eigenvectors are obtained through iterative methods that repeatedly multiply by the genotype matrix, as well as polygenic risk score computation and LD score regression, which project the genotype matrix onto a precomputed or random vector. Of these, PCA and mixed-model association via BOLT-LMM are the most demanding: both are widely used and statistically standard, and both run many iterations, so the per-product cost compounds and this primitive is the limiting factor.

The genotype representation graph (GRG) was recently introduced as a compact substrate for biobank-scale genotype data [6, 9, 12], achieving more than 25× compression relative to VCF.GZ on the UK Biobank WGS dataset, which includes 490,541 samples and 706,556,181 variants across 22 chromosomes. A GRG is a directed acyclic graph that captures genetic similarity among samples through shared ancestry-bearing nodes, so highly similar samples reuse a common representation of the variants they share. A GRG is constructed from haploid or phased genomes but supports both haploid and diploid computation. Because central operations such as allele frequency computation, genotype–phenotype association, and phenotype simulation run directly on the GRG via graph traversal that reuses computed values across shared nodes, they run orders of magnitude faster than the equivalent operations on tabular data [6, 9, 12]. GRG has demonstrated substantial gains in compressing and querying genotype data, but as cohorts grow to millions of individuals and analyses expand across many traits, SNPs, and environmental variables, further speed and portability to modern, rapidly evolving hardware are critical.

GPUs deliver much higher throughput and bandwidth than CPUs by using tens of thousands of cores [13], with more than 100-fold speedups reported for data analytics and machine learning [14–19]. SAIGE-GPU achieves a fivefold speedup over CPU-based SAIGE across more than two thousand phenotypes by porting the existing linear algebra to the GPU without changing the underlying genotype representation [20]. Unlocking full GPU performance, however, requires a parallel-friendly approach tailored to the task. GRG’s graph-structured reuse of shared relatedness does not map onto GPU hardware as naturally as a tabular format, so realizing its compression advantages as GPU throughput requires redesigning the computation. GRG traversal maps poorly onto GPU execution because node values must be resolved in strict topological order, which effectively serializes the dependency structure. Meanwhile, the skewed out-degree distribution and irregular, low-locality memory accesses further degrade load balancing, warp occupancy, and effective memory bandwidth.

GPU architectures are a natural fit for dense, regular computation, but substantial progress has also made sparse computation efficient on them, directly benefiting GRG-native computation, which is inherently sparse. Prior work [16, 17, 21–24] has mapped computational biology problems to sparse linear algebra primitives to enable acceleration. Such primitives, such as sparse matrix–vector multiplication (SpMV) [25, 26], are supported by highly optimized, portable libraries like cuSPARSE [27] and rocSPARSE [28], which hardware vendors and the research community maintain as architectures rapidly evolve. Our key observation is that sparse linear algebra primitives can bridge the gap between GRG traversal and efficient accelerator-based computing.

Methodologically, Mikado reformulates GRG traversal as a blocked sparse triangular solve built from sparse matrix-vector products, using wavefront scheduling that exposes the parallelism accelerators need while preserving GRG compression. The name reflects this scheduling rule, where, as in the game, a block is dispatched only after every block it depends on has been resolved, so computation advances as dependencies are satisfied rather than waiting at a global synchronization barrier. That formulation is implemented as a high-performance, hardware-portable CPU and GPU backend for grapp [9], relying only on standard sparse primitives so it carries across accelerator architectures without requiring reimplementation.

Mikado makes analyses that are practically infeasible with standard tools routine on commodity accelerator architectures. On the *All of Us* cohort [1], PCA that PLINK2 cannot complete on a single full chromosome within 72 hours finishes *in seconds*, and mixed-model association with BOLT-LMM-inf runs end to end orders of magnitude faster than with the official BOLT-LMM distribution. Relative to the GRG approach on which it builds, Mikado accelerates the core matrix–vector primitive by up to 470× and achieves order-of-magnitude end-to-end speedup and cost reduction for both analyses at biobank scale. These gains hold from cloud instances to supercomputer nodes. Beyond speeding up established analyses, lowering the cost of this primitive also makes it feasible to run analyses that were previously computationally impractical, such as exhaustive interaction scans across many traits and exposures. Benchmarks span simulated cohorts and the *All of Us* Research Program [1], demonstrating gains on real-world data at biobank scale.

Mikado uses the construction toolkit from prior work [6, 9] to build its GRGs and focuses on computing over them. Our contribution is a methodology that reformulates computation on GRGs as sparse linear algebra: (i) a reverse topological ordering that makes the adjacency matrix strictly block-lower-triangular with binary reachability, recasting the genotype matrix–vector product as a sparse triangular solve suitable for massively parallel architectures like GPUs; (ii) a pipelined wavefront schedule that decomposes the solve into overlapping blocked SpMV; and (iii) a hardware-portable CPU and GPU backend using standard vendor primitives, with the first GRG-native BOLT-LMM-inf formulation.

More broadly, the sparse triangular framing is a property of the representation’s structure rather than of the GRG specifically: any DAG-structured genotype encoding admits the same reverse topological ordering and block triangular solve. Our implementation realizes this framing concretely within the GRG ecosystem, and the same two-step reformulation applies to other DAG-based encoding schemes.

## 2 Results

The central computational primitive in statistical genetic analyses is the genotype matrix–vector product. Given the genotype matrix $G\in \{0, 1{\}}^{n\times m}$, where n is the number of haploid genomes and m is the number of mutations, the goal is to compute Gv for $v\in R^{m}$ or $G^{⊤}w$ for $w\in R^{n}$. Converting haploid Gv and $G^{⊤}w$ into corresponding diploid results is trivial [12]. Conventional approaches operate on an explicit genotype matrix, whether bit-packed and dense, as in the .bed format in PLINK1, or stored in a sparse representation, as in the .pgen format in PLINK2. In GRG-based computation, G is never explicitly materialized; it remains implicit in the graph’s reachability structure. In this section, we demonstrate that GRG matrix multiplication can be implemented efficiently using sparse block triangular solves (SpTRSV), that SpTRSV can be parallelized with SpMV-based block forward/backward substitution, and that even more parallelism can be achieved with a pipelined wavefront schedule.

### 2.1 From GRG Matrix Multiply to Block Sparse Matrix-Vector Multiply

Rather than formulating the product through whole-GRG traversal [6], these products can be expressed directly as sparse linear algebra primitives on the GRG adjacency matrix. Because the GRG is a directed acyclic graph (DAG), its nodes admit a reverse topological ordering, where every node precedes its ancestors. Under any such ordering, the adjacency matrix A is strictly lower triangular, and the genotype matrix is a submatrix of the transpose of the reachability matrix, $T^{⊤}=(I-A{)}^{-⊤}$. This follows from the definition of a GRG: an individual carries a mutation if and only if the corresponding mutation node can reach that individual’s sample node, so the reachability matrix encodes the genotype relation exactly.

Concretely, the genotype matrix–vector product decomposes as

(1) $$Gv=S\times \left(T^{⊤}\times \left(M^{⊤}v\right)\right),\;G^{⊤}w=M\times \left(T\times \left(S^{⊤}w\right)\right),$$

where S and M are selector matrices that map between graph nodes and samples or mutations, respectively. The selector multiplications are inexpensive scatter and gather steps; the actual work is the solve in between. The computational core applies $T=(I-A{)}^{-1}$ or its transpose to a vector: a sparse lower-triangular solve (SpTRSV) by forward substitution for $G^{⊤}w$, and the corresponding upper-triangular solve by backward substitution for Gv. For diploid analyses, the mapping between haplotype rows and per-individual genotype dosages is a linear operation absorbed into S and $S^{⊤}$, so no extra passes over the graph are needed in either direction.

For general DAGs, the entries in T can be any natural number. However, because GRG is a multi-tree DAG [6], there is at most one path between any two nodes, so T is guaranteed to be binary, which is consistent with G being binary. In addition, since A is binary, we can avoid materializing the values for the nonzero elements during SpTRSV using virtual memory (Methods).

Our reverse topological ordering is based on node *height*, defined for a node in a DAG as the length of its longest path to a leaf node. Ordering nodes by height gives a valid reverse topological ordering, since an edge from u to v requires u to have strictly greater height than v. Any such ordering is called a *level-set ordering*, following prior SpTRSV work [29, 30]. The ordering is not unique, as nodes of equal height may be arranged freely; that flexibility is used below to recover locality. Partitioning nodes by height gives H+1 levels, where H is the graph diameter, the longest shortest-path between any two vertices (typically H~30 for biobank-scale GRGs). Under any level-set ordering, A inherits a (H+1)×(H+1) block-triangular structure, meaning edges run only from higher to lower levels. Because level sets in GRGs are sparsely connected, each block of A is itself a sparse matrix, and the solve decomposes into a sequence of sparse matrix–vector multiplications (SpMVs) and additions. By treating each block as a unit, we turn the per-edge scalar operations into per-level-pair SpMVs. The forward substitution propagates values up the graph for $G^{⊤}w$, and backward substitution propagates them down the graph for $G_{v}$.

Each SpMV within a level is independent and embarrassingly parallel, so the number of sequential stages equals the number of levels. This is the level-set approach to SpTRSV [29, 30], with our height-based ordering as the analysis phase and block forward/backward substitution as the solve phase. The level-set ordering attains the minimum possible number of sequential stages, H, which is equal to the length of the critical path. Thanks to the multi-pass construction of the GRG, H grows only as $\sim {\text{log}}_{2}\;n$ with cohort size n. As a result, the block structure reduces the sequential depth from Θ(K), incurred by scalar forward/backward substitution that ignores block structure, to $\Theta (H)\sim \Theta \left({\text{log}}_{2}\;n\right)\leq \Theta \left({\text{log}}_{2}\;K\right)$.

Other reverse topological orderings expose less parallelism; the GRGL baseline, for instance, uses a postorder DFS labeling, which is itself a valid reverse topological ordering that favors single-threaded cache locality but can leave many nodes on a serial chain, which is a poor fit for GPUs (Methods). To maximize locality in our level-set ordering without compromising parallelism, we break ties among equal-height nodes by comparing their postorder DFS labels. Empirically, this gives the blocks of A a sparse structure that makes SpMV efficient (Methods). Finally, writing $A_{km}$ for the block that maps source level m to destination level k, this block can be dispatched as soon as level m is finalized, rather than waiting for all levels ℓ≤k-1. Mikado, our methodology and its CPU and GPU implementation, exploits this with a pipelined wavefront schedule that combines the simplicity of level-set methods [29, 30] and the parallelism of synchronization-free approaches [31]. On CPUs, it uses sparse routines from the Intel Math Kernel Library [32]; on GPUs, it uses sparse routines from cuSPARSE [27]. The block-triangular solve is described in full in Methods.

### 2.2 Kernel-Level Performance

The matrix–vector primitive comes first and sits at the core of downstream computation. On an NVIDIA A100 80 GB GPU, across five *All of Us* chromosomes at k=1, Mikado GPU achieves geometric-mean speedup of 452× for upward multiplication $\left(G^{⊤}w\right)$ and 326× for downward multiplication (Gv) over the single-threaded CPU GRG baseline [6], with a peak speedup of 473× for upward multiplication (Fig. 2a,b). The optimized single-threaded Mikado CPU backend achieves geometric mean speedups of 2.3× and 1.4× over GRGL in the upward and downward directions, respectively.

To provide additional context, we include two other GPU baselines: (i) a cuSPARSE SpTRSV implementation using the cusparseSpSV API, and (ii) a hand-written, graph-first GPU implementation with the optimizations described in Section 4. On the simulated 500k cohort, Mikado GPU achieves geometric mean speedups of 2.8× upward and 1.3× downward over direct SpTRSV, and ≈3.2× in both directions over graph first (Fig. 2c,d). Direct SpTRSV requires a separate external buffer for each direction, each at least as large as the sparse matrix itself, which leads to a memory cost of more than 2×. The other approaches, including Mikado GPU, reuse a single format for both directions to reduce memory usage. Given the same additional memory, they could apply further optimization, for instance by explicitly materializing the transposed matrix.

On *All of Us* chromosome 11, Mikado GPU is the fastest at every tested right-hand side (RHS) width (Fig. 2e,f). The scaling from k=1 to k=2 is sublinear, however, because the underlying cusparseSpMM API selects a specialized kernel for k=1. GRGL overtakes the optimized single-threaded Mikado CPU backend for downward multiplication at k=16, taking 53.3 seconds versus 74.8 seconds. Fig. 2g,h shows Mikado CPU performance with different thread and optimization configurations. Going from 1 to 16 threads without MKL optimization gives a geometric-mean upward speedup of 4.3× but essentially no downward speedup; optimization enables downward scaling but slows single-threaded downward execution.

Results on two additional machines, with NVIDIA V100 and H100 GPUs respectively, further demonstrate the efficiency and portability of Mikado’s formulation on GPUs (Extended Fig. 1). The end-to-end runtime benefits from these kernel-level gains in proportion to the fraction of total time the primitive consumes. This fraction is large for the iterative analyses below and small for single-pass workloads such as GWAS (Figs. 3, 4, and Extended Fig 3).

### 2.3 Biobank-Scale Analysis Applications

Mikado accelerates biobank-scale genotype matrix–vector products by up to 470× over the single-threaded CPU GRGL baseline [6], and this primitive-level gain translates into large end-to-end speedups across three widely used analyses. The end-to-end speedup depends on the proportion of runtime spent in matrix–vector products, since non-kernel work such as I/O and preprocessing is unaffected by the accelerated primitive; the larger that proportion is, the greater the end-to-end gain.

#### 2.3.1 Principal Component Analysis (PCA)

PCA extracts orthogonal directions of maximum variance and is widely used in population genetics to characterize ancestry and control for population stratification. On biobank-scale, randomized PCA is typically used, which reduces to repeated genotype matrix–vector products (Methods). On the full *All of Us* v8 phased cohort, Mikado GPU computes the top 10 principal components in 180 s at $1.00 per run ($0.14 compute-only), compared with 5,391 s at $4.72 ($4.52 compute-only) for GRGL, making it roughly 30× faster and 4.7× cheaper (Fig. 3b). The simulated 200,000- and 500,000-individual cohorts show roughly 10× over GRGL, while PLINK2 completes PCA only on 1000 Genomes, where it is 278× slower than Mikado GPU. Mikado with MKL offers only marginal improvement over GRGL because the multi-chromosome workload is memory-bound and the formulation does not reduce the number of memory accesses.

For single-chromosome experiments, Mikado achieves larger speedups over GRGL: up to 71× on GPU and 6.9× with MKL (Fig. 3a). GRGL does not support multithreading within a single chromosome, whereas Mikado’s formulation can exploit hardware parallelism. PLINK2 cannot complete PCA on any full chromosome within a 72-hour limit; on filtered chromosome 21, it finishes in 36,452 seconds, against which Mikado achieves a 3648× speedup on GPU and a 1132× cost reduction. These gains come from (i) GRG’s compression, which reduces both memory footprint and arithmetic work, and (ii) Mikado’s formulation, the contribution of this work, which maps that computation onto optimized kernels that exploit the parallelism and memory bandwidth of modern GPUs.

Under the block-vector LOBPCG solver, Mikado GPU consistently outperforms GRGL, while Mikado CPU’s advantage shrinks as the number of principal components increases, in line with the kernel benchmark (Fig. 3c). Unlike the default eigsh solver, which multiplies the GRG by a single vector at each step, LOBPCG multiplies it by a block of up to $n_{\text{PC}}$ columns. Therefore, this panel shows backend performance for applications that benefit from block multiplications.

#### 2.3.2 BOLT-LMM Infinitesimal (BOLT-LMM-inf)

BOLT-LMM-inf [10] is the infinitesimal variant of BOLT-LMM, widely used for biobank-scale GWAS in settings where unmodeled relatedness or population structure would otherwise inflate association test statistics. Its heritability estimation and per-variant calibration stages both rely on a conjugate gradient solver that invokes the matrix–vector primitive once per iteration (Methods), making BOLT-LMM-inf the most MVP-intensive of the three applications in this work.

Our evaluation runs BOLT-LMM-inf on the 22 chromosomes from the 1000 Genomes Project [33], chromosomes 19–22 from the simulated 200,000-individual cohort, and the 22 chromosomes from the full *All of Us* v8 phased cohort (Fig. 4a). In the simulated cohort, which is the only dataset with a synthetic ground truth, the estimated heritability is $\hat{h^{2}}=0.579444$ for official BOLT-LMM and $\hat{h^{2}}=0.577319$ for the Mikado GPU implementation, compared with the ground truth value $h^{2}=0.6$. Close agreement between the two estimates indicates that Mikado GPU preserves the statistical power of downstream applications, consistent with GRG being a lossless representation. Furthermore, the $\chi^{2}$ test statistics from the Mikado implementation match the official BOLT-LMM v2.5 values up to a global multiplicative constant, which is attributable to differences in the pseudorandom number generator (PRNG) implementation used for Monte Carlo trace estimation and calibration-SNP selection (Extended Data Fig. 4, Supplementary Note 4.6).

For 1000 Genomes, Mikado GPU completes in 183.3 s, compared with 4,742.0 s for the BOLT-LMM reference baseline, a 25.9× end-to-end speedup, whereas GRGL finishes in 2,737.4 s, a 1.7× speedup over BOLT-LMM (Fig. 4a). For the four-chromosome experiment on the simulated 200,000-individual cohort, the gap between Mikado GPU and BOLT-LMM increases to 1,480.3×, while GRGL also improves to 19.4×, driven by GRGL’s sublinear growth rate. On the full *All of Us* cohort, where BOLT-LMM becomes prohibitively expensive to run, Mikado GPU and CPU achieve 18.7× and 1.1× speedup over the GRGL baseline, respectively. For cost-effectiveness on *All of Us*, Mikado GPU on an A100 instance is 18.7× faster at 1.6× the hourly cost, yielding 11.7× higher overall cost-effectiveness than GRGL (Fig. 4a). Because Mikado CPU uses the same hardware as GRGL, its 1.1× speedup translates directly into 1.1× more work per dollar.

## 3 Discussion

Recasting GRG traversal as sparse linear algebra changes what biobank-scale population genetics can achieve and on what budget. The reformulation relies on the fact that, under a topological ordering, the GRG adjacency matrix A is strictly lower triangular. Consequently, the genotype matrix–vector product becomes a sparse triangular solve that decomposes into a pipelined sequence of blocked SpMVs, with one block per height level of the graph and depth bounded by the graph’s diameter. This primitive has broad impact because a wide range of population genetics computations can be reduced to it. Randomized and truncated PCA reduce to repeated genotype matrix–vector multiplies against the standardized genotype matrix [34, 35]; mixed-model association solvers such as BOLT-LMM invoke the same primitive within their conjugate-gradient iterations [10]; and model-based ancestry estimators, including ADMIXTURE [36, 37], alternate updates dominated by products of the genotype matrix, or its transpose, with the parameter vector. Even sparse nonnegative matrix factorization methods like sNMF [38], which rely on matrix–matrix multiplication, can benefit from a fast matrix–vector primitive by iterating over the columns of the right-hand-side matrix. Because such matrix–matrix products are typically compute-bound, Mikado greatly reduces the required arithmetic operations by sharing intermediate values across graph nodes. Thus, any method built on this primitive inherits Mikado’s throughput and cost advantages by substituting the GRG-backed version for its dense or PLINK2-based counterpart, without changing the surrounding statistical model. By accelerating this shared primitive, Mikado reduces multi-day PLINK2 PCA and BOLT-LMM runs to minutes or seconds, further improving on the CPU-only GRG method it builds on.

Methodologically, expressing the computation as a standard primitive lets the approach inherit performance and portability from a rapidly evolving accelerator software stack, such as cuSPARSE [27], without ongoing custom kernel engineering. This matters because the computing and accelerator ecosystem is now changing rapidly [39], and hand-maintained code is a recurring liability and effort, not a one-time cost. Relying only on standard sparse BLAS, Mikado’s implementation runs on different hardware without accelerator-specific reimplementation, with end-to-end results reported on both a cloud configuration (*All of Us* Researcher Workbench on Google Cloud, using both GPU and CPU instances) and the Perlmutter supercomputer at NERSC. The same code runs across NVIDIA GPU models through cuSPARSE and can extend to non-NVIDIA accelerators through libraries such as ROCm and rocSPARSE, and GRG construction is inherited unchanged from the original toolkit [6]. This span ranges from discrete PCIe-attached accelerators such as the A100 to tightly coupled Grace Hopper machines such as the GH200 superchip (Extended Fig. 2). Because Mikado is expressed in standard primitives rather than a bespoke kernel, the mature sparse triangular solve (SpTRSV) literature [40–42] becomes directly applicable, making the adoption of a faster scheduler a drop-in improvement rather than a redesign.

On a single GPU the core product accelerates by 470× over the CPU GRGL baseline [9], but the end-to-end speedup is governed by the fraction of runtime spent on matrix–vector work. This fraction defines a spectrum. Computations with few matrix–vector multiplies sit at the low end, dominated by chromosome loading and improving marginally, whereas PCA and BOLT-LMM-inf place the matrix–vector multiply in an iterative inner loop and achieve orders-of-magnitude end-to-end speedup. Operating on cloud infrastructures required for biobank data, such as *All of Us*, makes cost a first-class concern, not just runtime, since the same budget then enables more analyses. Because Mikado finishes much sooner, the shorter runtime offsets any hourly-rate premium, so accelerated analysis is typically both faster and cheaper; the two ratios vary by workload. Our focus is on the ratio of time and cost saved rather than the absolute values, because that ratio carries over to other scenarios. For example, a study spanning thousands of phenotypes, G×E environments, or LOCO segments would inherit these savings, multiplied across every run. Big data and machine learning research already benefit in speed and cost from modern accelerators; Mikado extends those advantages to population genetics, where irregularity and sparsity have kept them out of reach.

The reformulation is not specific to the GRG. In fact, any DAG-structured genotype representation admits the same sparse triangular framing, so the performance and portability advantages transfer. The linear ARG [43, 44] is a closely related encoding whose authors observed its equivalence to the triangular system but pursued a graph-traversal solver instead; the two-step reformulation described here applies to it directly. Because it constructs a general DAG rather than a multi-tree, diamond-shaped paths introduce negative edges, requiring general SpTRSV rather than binary SpTRSV. More broadly, efforts such as traceax [45], a JAX-based [46] framework for stochastic trace estimation, accelerate matrix–vector multiplication on an explicit genotype matrix; the approach here instead accelerates the product implicitly on the compressed GRG, so any downstream estimator, trace-based or not, inherits the speedup.

The main limitation of Mikado is the device memory ceiling: single-node analysis is limited to cohorts whose per-chromosome GRGs fit within the total GPU memory available. A host-memory streaming mode that transfers blocks on demand (Methods), as well as partitioning across nodes for larger cohort sizes, are possible routes past this ceiling. A secondary cost is memory footprint, since the block format roughly doubles the stored size relative to GRGL’s delta encoding, which compresses repeated edges (Methods); this overhead is removable rather than intrinsic. Delta encoding can also be applied to the block format, recovering compression while retaining the traversal structure, a direction left for future work.

The scientific impact of removing this compute bottleneck is that analyses become feasible, not just faster, as illustrated by the PLINK2 and BOLT-LMM comparisons above. Under a fixed computational budget, an order-of-magnitude reduction in per-analysis cost translates into a correspondingly larger effective sample size or a broader set of tested models, which matters most when computation is the binding constraint. Gene-environment interaction is the clearest case: the number of tests grows multiplicatively because each variant is tested against each exposure, so researchers typically limit analysis to a few exposures and a few traits [47]. Researchers can now scan many exposures across many traits, making exhaustive interaction scans possible at biobank scale and helping identify new interactions. In addition, gene-gene interaction tests are typically performed on a restricted set of genetic loci [48, 49], which could miss true interactions. Most importantly, a cheap, portable genotype matrix product scales existing approaches, such as BOLT-LMM, to previously unimaginable cohort sizes. By lowering the cost of the underlying operation, it enables practical analyses that were previously infeasible, including scans for three-way interactions among genetic and environmental factors, whether between two genes and an environmental exposure or among three genes.

## 4 Methods

**Algorithm 1:** Level-set Block Forward Substitution

**Require:** $A\in R^{K\times K}$, cutoffs ${\left\{c_{k}\right\}}_{k=0}^{H+1},x\in R^{K}$
**Ensure:** $y\in R^{K}$ such that $y=(I-A{)}^{-1}x$
1: y←x
2: **for** k=0 **to** H **do**
3: $I_{k}\leftarrow \left[c_{k},c_{k+1}\right)$
4: **for** m=0 **to** k-1 **in parallel do**
5: $I_{m}\leftarrow \left[c_{m},c_{m+1}\right)$
6: $y_{I_{k}}\leftarrow y_{I_{k}}+A_{I_{k},I_{m}}y_{I_{m}}$
7: **end for**
8: **end for**


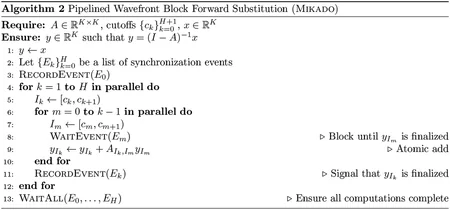


### 4.1 Genotype Representation Graph

A GRG encodes genotypes as a directed acyclic graph with a multi-tree structure [6, 9]. A multi-tree is a directed acyclic graph (DAG) in which a node may have multiple parents, but at most one directed path connects any pair of nodes. The samples are mapped to leaf nodes, while variants may be mapped to any node in the graph. Each variant allele is associated with a node, and paths connect samples to the variants they carry. Sample $s_{i}$ carries variant $v_{j}$ if and only if the node associated with $s_{i}$ is reachable from the node associated with $v_{j}$. For example, in Fig. 1a, the sample at node 1 carries exactly mutations $m_{0}$ and $m_{1}$ (from node 2), $m_{2}$ (from node 4), and $m_{5}$ (from node 1 itself); it does not carry $m_{4}$ because no path leads from node 8, which carries $m_{4}$, to node 1. This structure allows a GRG to compress genotype datasets losslessly, and GRGs can be constructed efficiently from explicit, dense genotype matrices [6, 9].

Beyond compression, the GRG enables fast computation directly on the genotype matrix without first decompressing it. That is, multiplying the genotype matrix by a vector is equivalent to a downward traversal of the GRG, where the input vector supplies initial values at the mutation nodes. Conversely, multiplying by the transposed genotype matrix corresponds to an upward traversal, where the leaf nodes are assigned the initial values. The compression reduces both memory footprint and computation, because shared subgraphs are visited once rather than once per sample.

### 4.2 Node Ordering and Wavefront Scheduling

Our SpTRSV formulation works for any reverse topological ordering, but the specific choice trades off parallelism against locality. The baseline GRGL toolkit uses postorder DFS labeling, which is a valid reverse topological order. It keeps dependent nodes close for cache reuse but leaves many blocks with only a single node, which limits parallelism. Equation 2 highlights the block structure that partitions A into maximal contiguous dependency-free batches: postorder DFS yields five blocks and four sequential steps (left), whereas any level-set ordering yields only three blocks and two sequential steps (right). The former favors temporal locality; the latter favors parallelism, at the cost of cache thrashing on a machine whose cache can hold only one node at a time.


(2)

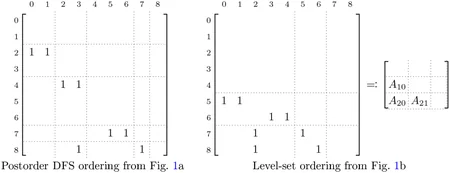



Level-set ordering minimizes the number of sequential steps, making it well suited to massively parallel accelerators like GPUs. To approach the parallelism–locality Pareto frontier, we recover locality without increasing the sequential depth by exploiting the freedom in within-level ordering: ties among equal-height nodes are broken using their postorder DFS labeling. In practice, constructed .grg files already carry postorder DFS labels, so Mikado only needs to stably sort nodes by height. This within-level ordering keeps each node’s dependencies close. In Fig. 1b, node 5 depends on nearby nodes 0 and 1 rather than on distant ones. The dependencies of adjacent nodes also stay close to one another: if Fig. 1b were a sub-DAG of a larger graph with many height-zero nodes, nodes 5 and 6 might be labeled 1005 and 1006, but their dependencies (0 and 1, and 3 and 4) would still be adjacent. Empirically, some blocks of A exhibit a nonzero diagonal pattern, improving cache locality during SpMV.

The chosen ordering reduces the genotype matrix–vector product to the block-triangular system (I-A)x=y. For the three-level example in Fig. 1c, this is:

(3) $$\left[\begin{array}{ccc} I & \; & \;\\ -A_{10} & I & \;\\ -A_{20} & -A_{21} & I \end{array}\right]x=y,$$

solved by block forward substitution: $x_{0}\leftarrow y_{0}$, then $x_{1}\leftarrow y_{1}+A_{10}x_{0}$, then $x_{2}\leftarrow y_{2}+A_{20}x_{0}+A_{21}x_{1}$. A level-set schedule places a global barrier between these steps, so level k begins only after every node in levels 0,…,k-1 has been finalized (Algorithm 1). Our wavefront schedule (Algorithm 2) removes the barrier: each summand $A_{km}x_{m}$ is dispatched as soon as its source level m is finalized, so $A_{20}x_{0}$ proceeds without waiting for $x_{1}$, and independent blocks overlap between levels. The savings are shown as the shaded interval in Fig. 1d.

The parallelism-locality tension, and the DAG scheduling problem more broadly, are well studied [50, 51]. Because Mikado recasts GRG traversal as standard sparse BLAS, the mature SpTRSV literature applies directly. For example, existing schedulers such as SpMP [40], DAGP [41, 42, 52], and HDagg [42], along with recent advances like GrowLocal [53], DFA-SpTRSV [54], and ElasticDivide [55], are drop-in improvements rather than complete redesigns.

### 4.3 Mikado’s Implementation

For Mikado CPU, Algorithm 1 was implemented using the inspector-executor API for sparse BLAS routines in Intel MKL [32]. The vendor SpMV routines require a matrix handle in a standard sparse format (CSC, CSR, or COO). Following GRGL’s convention, we store A in CSR format, or equivalently, $A^{⊤}$ in CSC format. Since all nonzero entries of our blocks $A_{I_{k},I_{m}}$ equal one, the val array is redundant. This redundancy was exploited by allocating a shared all-ones array, aliasing it to the val pointer of every block, and mmaping it to span arbitrarily many logical bytes while occupying few physical bytes, greatly reducing the handle’s memory footprint. MKL’s optional optimize API analyzes matrix structure and applies hint-based optimizations [32], but it allocates an internal buffer roughly the size of the original matrix and is incompatible with the aliasing scheme, at least doubling memory usage; it is therefore exposed as a user option. MKL’s multithreaded single-matrix SpMV scaled unpredictably, especially with optimization disabled, so chromosome-level parallelism was prioritized following GRGL, with multiple chromosomes processed concurrently across threads. Hybrid parallelism is supported, and allocating more threads to larger chromosomes helps mitigate the straggler effect caused by load imbalance in some cases.

On GPU, two strategies implement GRG-based matrix–vector products: a graph-first baseline that ports the CPU traversal logic to the GPU, and the Mikado GPU approach, the primary contribution of this work. Both target NVIDIA GPUs, use CUDA streams, share the matrix-vector primitive used by the applications, and inherit GRG construction unchanged from the original toolkit [6]. The graph-first baseline mirrors CPU GRG traversal: nodes were partitioned by height to break dependencies, and a height-level kernel propagated values between adjacent levels using warp-level reductions for bottom-up traversal and atomicAdd for top-down traversal. It was optimized in three ways:

1. To amortize per-block scheduling overhead, each thread block processed multiple same-level nodes when k=1. For larger k, each thread block handled a single node, with consecutive threads assigned to the same down edge for coalesced access;
2. For k≠1, a node’s edges were staged in a shared memory buffer before accessing their values, overlapping edge loading with computation;
3. Threads per node were assigned dynamically to balance the load within each height partition, with a dedicated outlier kernel giving high-degree nodes extra threads.

This baseline depends heavily on CUDA and cannot be retargeted directly, but it is already substantially faster than the CPU implementation [6] (Fig. 2c,d).

The Mikado GPU approach instead expresses the computation as a sequence of standard sparse primitives. The adjacency matrix A was stored once in a reverse topological (level-set) ordering that makes it strictly lower triangular, partitioned into height-based blocks $A_{I_{k},I_{m}}$, and Algorithm 2 was implemented by dispatching off-diagonal blocks to cusparseSpMM routines running in concurrent CUDA streams, orchestrated with CUDA events. The virtual memory aliasing described above was applied on the device using the CUDA Virtual Memory Management API. For datasets whose GRGs exceed a single GPU’s memory, entire set of per-chromosome GRGs were assigned to separate devices via a device map rather than partitioning a single GRG. NVLink is used when available, since GPU-to-GPU communication can otherwise dominate runtime. For GRGs too large to fit in device memory, a streaming mode transfers blocks on demand from host memory using double buffering, so one block is processed while the next is in transit (Extended Fig. 2). Because Mikado relies only on standard sparse BLAS, it can be retargeted across hardware without vendor-specific rewrites, including non-NVIDIA GPUs, whereas the graph-first baseline, written against CUDA, cannot be retargeted without substantial reengineering.

The block formulation reorganizes the nodes and edges of the native GRG format into a block format, a lightweight conversion that requires no significant computation. Because the converted .grg_spmv format does not yet support delta encoding, its files are larger than the corresponding .grg files: on *All of Us*, .grg_spmv totaled 193 GB (1.9× the .grg size), yet was still 15.6× smaller than the corresponding .vcf.gz.

### 4.4 Per-Application Matrix-Vector Workload

The three applications differ in how heavily they exercise the matrix-vector primitive. GWAS performs two matrix-vector products per variant and is kernel-bound. PCA has two equivalent formulations: (i) an eigendecomposition of the genetic relationship matrix and (ii) a singular value decomposition of the genotype matrix. Critically, at biobank scale, exact eigendecomposition is infeasible, so randomized SVD or implicitly restarted Lanczos are used; both reduce PCA to repeated genotype matrix–vector products. The top 10 principal components of *All of Us* were computed with grapp using a SciPy/CuPy implicitly restarted Lanczos solver [9], and compared against GRGL and PLINK2 pca -approx, which uses a similar randomized block Lanczos method [2].

BOLT-LMM-inf [10] has two main steps: a restricted maximum likelihood (REML) subroutine for heritability estimation (step 1a) and per-variant calibration using leave-one-chromosome-out (LOCO; step 1b). Both rely on a conjugate gradient (CG) solver in sample space, $GG^{⊤}x=y$ with $x,y\in R^{n}$, invoking the matrix-vector primitive once per CG iteration. The total count is $\left.O\left(k_{\text{a}}ET+k_{\text{a}}(F+R)\right)\right)$, where $k_{a}$ is the average number of CG iterations during step 1a, E is the number of variance-parameter evaluations, T the Monte Carlo trials, $k_{b}$ is the average number of CG iterations during step 1b,, F the LOCO folds, and R the calibration SNPs. The infinitesimal model was chosen over the more complex spike-and-slab BOLT-LMM because (i) its $\chi^{2}$ statistics are essentially identical to GCTA-LOCO at the SNP level for polygenic traits, (ii) it is the statistically correct model for highly polygenic traits such as height, and (iii) full BOLT-LMM begins by running BOLT-LMM-inf, which accounts for ~40% of total runtime [10].

### 4.5 Experimental Setup

#### Computing Hardware

Our experiments ran primarily on the *All of Us* Researcher Workbench, hosted on Google Cloud. GPU experiments used A2 Ultra instances with NVIDIA A100 80 GiB SXM GPUs, with up to four per node connected via third-generation NVLink. For single-chromosome CPU experiments, we used n2-standard-32 instances (32 vCPUs, 16 physical cores, 128 GB); smaller instances are more susceptible to noisy neighbor interference from co-tenant workloads. For multi-chromosome experiments, we used n2-highmem-48 instances (48 vCPUs, 24 physical cores, 384 GB), with one physical core per chromosome and enough memory to hold the chromosomes concurrently. On-demand pricing at the time of writing was $1.56/hour (n2-standard-32), $3.15/hour (n2-highmem-48), $5.03/hour (single A100), and $20.12/hour (four A100s). Because these rates are metered, we report dollar cost along-side wall-clock time, as the two do not necessarily move together. A GPU instance runs at a higher hourly rate than a CPU instance but, because it finishes much sooner, it is often cheaper overall, so Mikado on GPU can often be both faster and cheaper than the CPU GRG baseline. On identical CPU hardware with a fixed hourly rate, cost savings match runtime savings exactly. However, since GRGL uses less memory than Mikado CPU, it is possible to switch to lower-tier machines and reduce costs. This depends on multiple factors, including memory bandwidth, which is usually not specified or guaranteed on cloud platforms. The Researcher Workbench does not allow local disks with controlled-tier data, so datasets reside in Cloud Storage buckets with lower bandwidth and higher network variability. To mitigate this, inputs were staged on a RAM disk for all methods when memory permitted, and staging time was excluded from the reported runtimes. The Perlmutter supercomputer was used for the 1000 Genomes [33] and simulated datasets. Each GPU node has one AMD EPYC 7763 (Milan) 64-core CPU, 256 GB DDR4 memory, and four A100 GPUs on third-generation NVLink, and was used for both CPU and GPU runs. In addition, the kernel runtimes are evaluated on DeltaAI with Grace Hopper GH200 superchips and on SDSC Expanse V100 nodes. Each GH200 superchip has a 72-core Grace ARM CPU with 120 GB of memory and an NVIDIA H100 GPU with 96 GB of memory. Each SDSC Expanse V100 node has two Intel Xeon 6248 CPUs, 384 GiB of memory, and four NVIDIA V100 SXM2 32 GiB GPUs. Every single-chromosome experiment uses one GPU; multi-chromosome experiments use either one or four GPUs, as noted for each experiment.

#### Computing Software

Mikado depends on two vendor math libraries: cuSPARSE and Intel MKL. On GPU, we use CUDA 13.3.1 with cuSPARSE 12.8.2.51; this version or newer is required, as earlier releases contain a bug that affects numerical precision. Because the *All of Us* Researcher Workbench provides only CUDA 12.8, we rely on CUDA forward compatibility to load newer libraries. For the CPU, we use Intel MKL 2026.1.

For PLINK, we use the PLINK2 alpha 6.9 LM 64-bit Intel build preinstalled on the *All of Us* Researcher Workbench, and PLINK2 alpha 7.1 64-bit AVX2 Intel on all other platforms. For BOLT-LMM, we built version 2.5 from source, linked against Intel MKL. GRGL 2.9 and a slightly modified version of grapp were used^2^.

### 4.6 Dataset, Quality Control, and Statistical Analysis

The primary dataset was the srWGS statistical phasing call set in the *All of Us* CDRv8 release, since phased data are recommended for GRG construction [6]: 414,830 individuals and 870,337,413 variants across 22 chromosomes. GRGs were constructed with GRG Library v2.9 [9] using default parameters^3^ Three supplementary datasets supported smaller-scale and controlled-scaling experiments. The 1000 Genomes dataset [33] is a smaller WGS cohort consisting of 3,202 individuals and 71,632,912 variants. For controlled scaling, two simulated cohorts of 200,000 and 500,000 individuals spanned all 22 autosomes at full GRCh38 length. These cohorts were generated under the four-population OutOfAfrica_4J17 demographic model with stdpopsim [56] and the msprime backend, drawing equal numbers of diploid individuals from CEU, CHB, JPT, and YRI.

Kernel benchmarks and GWAS analyses were run on unfiltered datasets. PCA was run unfiltered, except for filtered chromosome 21 from *All of Us* v8, where non-SNP and multiallelic variants were removed. The BOLT-LMM analyses used additional filtering applied per dataset. On *All of Us* v8, variants were filtered by minor allele frequency (MAF ≥ 5 × 10^−5^) and Hardy–Weinberg equilibrium $\left(p\geq 10^{-12}\right)$, with non-SNP and multiallelic sites removed; individuals were restricted to the EUR group in the *All of Us* ancestry file, excluding those with quality-control flags, leaving 220,760 individuals and 44,626,943 variants. 208,382 individuals with height statistics are used in the experiement. For the simulated 200K cohort (last four chromosomes), variants were filtered by MAF ≥ 10^−4^ and multiallelic sites removed. For 1000 Genomes, variants were filtered by MAF ≥ 10^−2^, with non-SNP and multiallelic sites were removed.

Both GWAS and BOLT-LMM require inputs beyond the genotype data. For GWAS, phenotypes were simulated at random. For BOLT-LMM on the simulated 200K cohort, phenotypes were generated with grg-pheno-sim v1.5 at heritability 0.6. On *All of Us*, we used standing height (concept ID 903133), including measured and self-reported values, with sex, age, and the top 10 principal components of the filtered subset as covariates.

The agreement with reference implementations was assessed without re-deriving the reference statistics. For GWAS, per-variant p-values above genome-wide significance were compared against PLINK2 on 1000 Genomes: across all variants, the GRG-based approach attains $R^{2}>0.999$ for every statistic, with maximum absolute error below ^10−5^. For PCA, the top 10 principal components were compared against PLINK2 and against grapp with the GRGL backend, using per-component cosine similarity with signs aligned to resolve eigenvector sign ambiguity; Mikado attains a cosine similarity of 1.000 with GRGL for all 10 components. PLINK2 produces unstable results for PC7–PC10 across runs on *All of Us*, so the PLINK2 comparison is restricted to the top six components, for which Mikado achieves a cosine similarity of 1.000. For BOLT-LMM-inf, per-SNP $\chi^{2}$ statistics were compared against the official BOLT-LMM output using the squared Pearson correlation. On both the 1000 Genomes dataset and the simulated 200K cohort (last four chromosomes), the GRG-based approach attains $R^{2}>0.999$. The timing comparisons report end-to-end wall-clock time under the staging convention above, with dollar cost based on the rates under **Computing Hardware**. For PLINK2, we use the default parameters for random seeds in PCA experiments. For GRG-based PCA, we use NumPy’s RNG with random seed random seed 0 to generate the initial vector. For BOLT-LMM, we use the random seed 2026. The experiments use float64 as the data type.

## Supplementary Material

1

**Supplementary Information**. Extended Figures and Supplementary Information are available for this paper.

## Figures and Tables

**Fig. 1:**
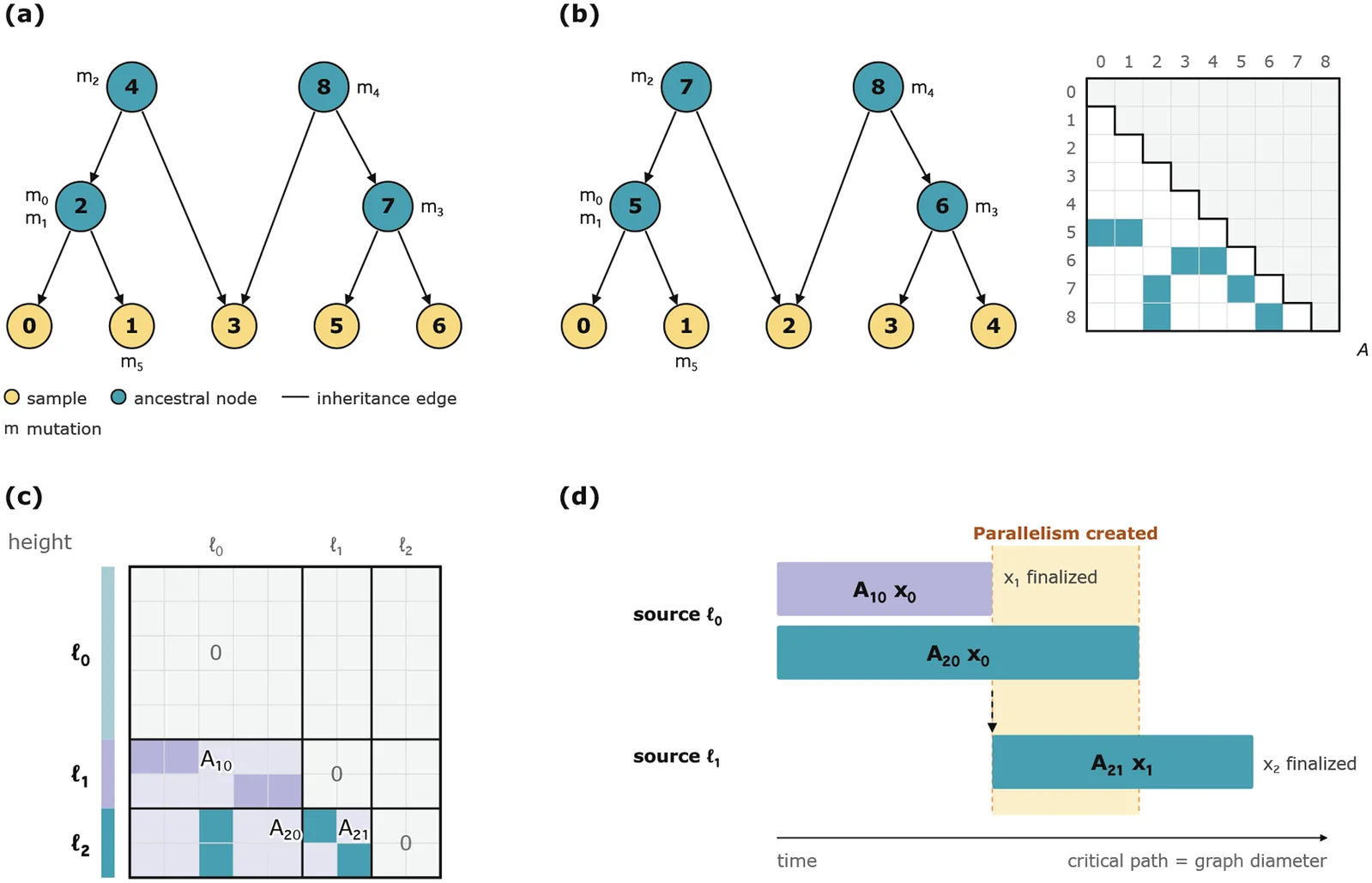
Our level-set relabeling transforms a GRG into a block-triangular matrix that exposes efficient pipelined execution. **(a)** A toy GRG with five samples (white) and four ancestral nodes (teal), carrying mutations $m_{0}-m_{3}$; edges point from ancestors to descendants. Nodes are numbered in the post-order produced during graph construction. **(b)** The same graph after level-set relabeling. Every edge now runs from a higher to a lower index, so the adjacency matrix A is strictly lower triangular; blue cells are ones and gray cells are structural zero entries. **(c)** Grouping nodes by height partitions A into blocks $\ell_{0},\ldots ,\ell_{2}$; purple cells are ones representing the down edges from nodes in $\ell_{1}$, blue cells are ones representing the down edges from nodes in $\ell_{2}$, and gray cells are structural zeros; no edges join nodes of equal height, so the diagonal blocks vanish and only the strictly lower blocks $A_{10},A_{20}$, and $A_{21}$ carry entries; each block is one internally parallel SpMV. **(d)** Our wavefront schedule. Rows indicate the source level supplying the input. The summand $A_{20}x_{0}$ for $x_{2}$ is dispatched as soon as $x_{0}$ is ready, without waiting for $x_{1}$ to finalize, so $A_{21}x_{1}$ begins the moment $x_{1}$ is ready while $A_{20}x_{0}$ is still running; the shaded interval is the time saved relative to a level-set schedule, which synchronizes at every level.

**Fig. 2:**
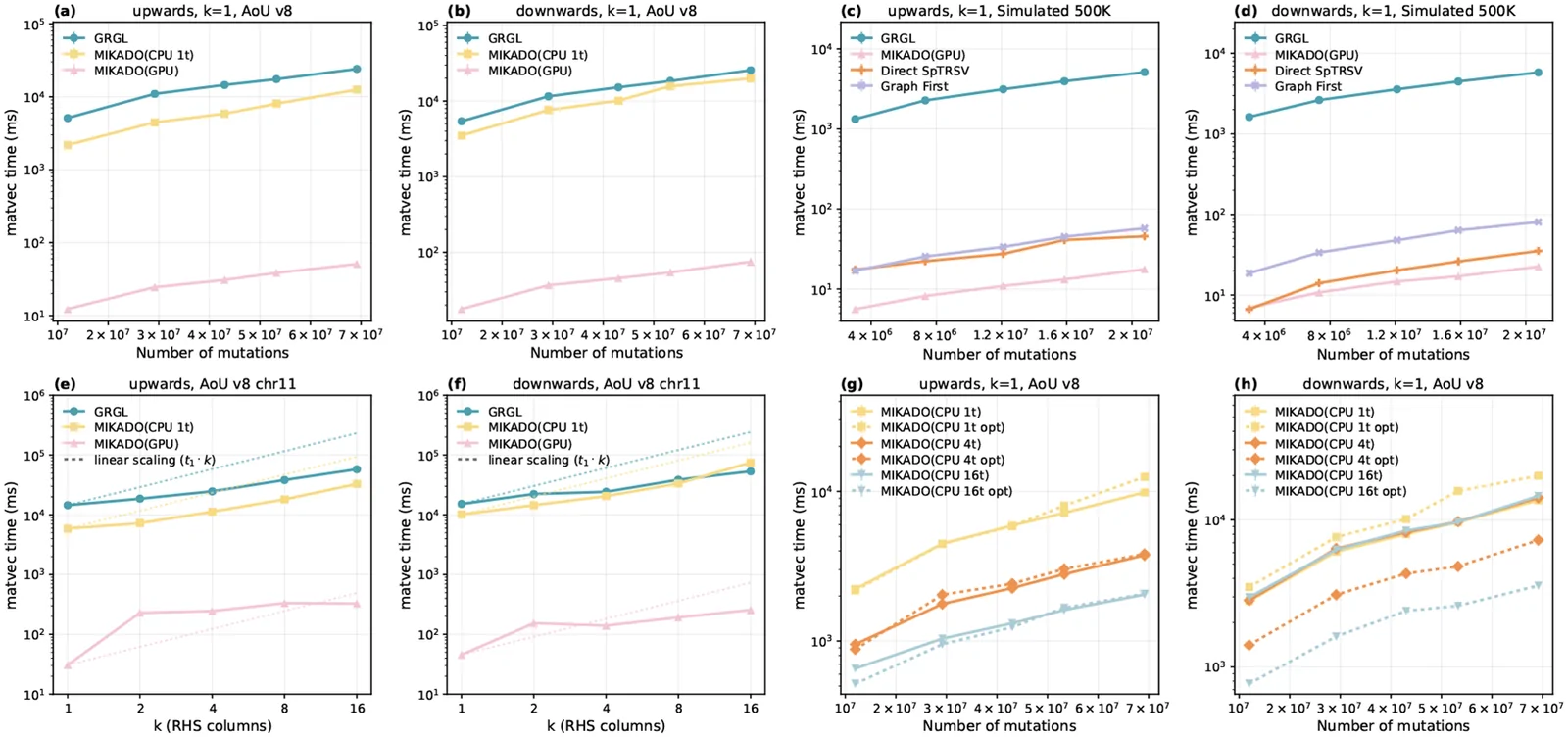
Performance of the matrix–vector primitive. Each panel pair shows upward (left) and downward (right) traversal. MKL’s optimize mechanism (Methods) is enabled for the Mikado CPU curves in **(a,b)** and **(e,f)**. **(a,b)**: Primitive runtime across five *All of Us* v8 chromosomes at k=1 for both upward and downward traversal. GRGL, Mikado CPU (with MKL), and Mikado GPU (with cuSPARSE) are shown. **(c,d)**: Primitive runtime on five chromosomes from the simulated 500k cohort at k=1. GRGL, Mikado GPU, and two additional GPU-based codes are chosen. Direct SpTRSV uses the SpSV primitive provided by cuSPARSE. Graph-first is a hand-written, highly optimized, traversal-based implementation (Methods); (e,f): Right-hand side (RHS) width scaling performance of GRGL and Mikado on *All of Us* v8 chromosome 11. Here, k is the number of RHS columns propagated per traversal. For Mikado GPU, increasing k from 1 to 2 multiplies runtime by 7.5 upward and 3.4 downward, whereas increasing k from 2 to 16 multiplies runtime by only 1.4 and 1.7, respectively. (g,h): Mikado CPU scaling on the same *All of Us* chromosomes at k=1, using 1, 4, and 16 threads, with and without optimization. Optimization requires preprocessing time and memory (Methods). For a fixed thread count, optimization yields geometric-mean downward speedups of 2.0× at four threads and 3.8× at 16 threads (3.5–4.1× across chromosomes), at the cost of a 24–63% higher single-thread runtime; upward benefits are small and variable.

**Fig. 3:**
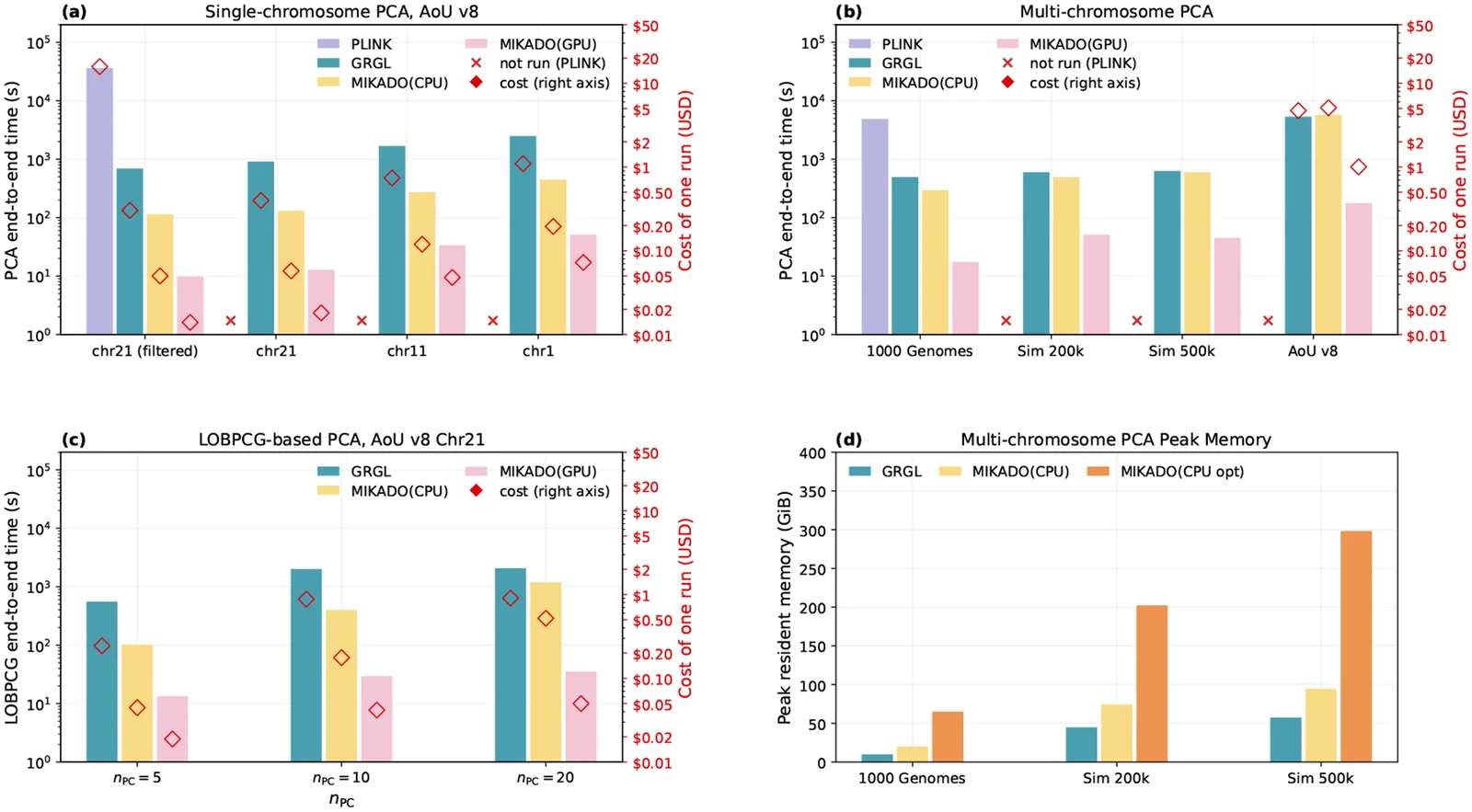
Runtime and cost of PCA and BOLT-LMM-inf. Time is shown with left axis. The corresponding cost on the Researcher Workbench is shown with right axis. **(a)** A single-chromosome PCA on *All of Us* v8. chr21(filtered) removes non-SNP and multiallelic variants. Mikado CPU and PLINK uses 16 threads. MKL’s optimize is enabled and optimization time is included. PLINK failed to compute results within the 72 hours time limit, except for chr21 (filtered). **(b)** Multi-chromosome PCA on four different datasets. Mikado CPU uses 1 thread per chromosome; Mikado GPU uses 4 GPUs. MKL’s optimize is disabled. PLINK failed to compute results within the time limit, except for 1000 Genomes. **(c)** Single-chromosome LOBPCG-based PCA on chr21 from *All of Us* v8. Mikado CPU uses 16 threads. MKL’s optimize is enabled. LOBPCG begins by multiplying the genotype matrix by a block of $n_{\text{PC}}$ columns; the block size decreases as individual eigenpairs converge and are deflated. **(d)** CPU memory usage for multi-chromosome PCA across GRG-based backends. GRGL uses the least memory. The Mikado CPU based on the MKL backend uses 1.6–2.0× as much memory, reflecting the absence of delta encoding in the .grg_spmv format. Mikado CPU memory usage increases to roughly 3× when MKL optimization is enabled.

**Fig. 4:**
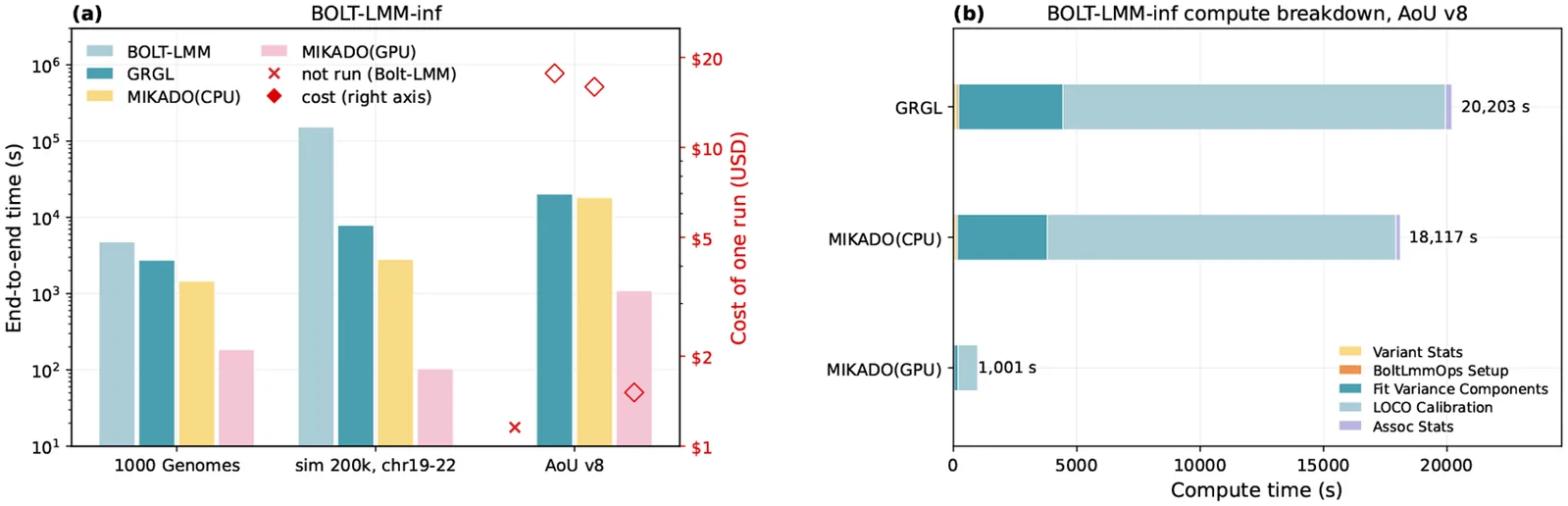
Runtime and cost of BOLT-LMM-inf. **(a)** The multi-chromosome BOLT-LMM-inf runs. The three datasets are further filtered. Output write time is excluded for all implementations. Mikado CPU uses 1 thread per chromosome; Mikado GPU uses 1 GPU. MKL’s optimize is disabled. The official BOLT-LMM is allowed 64 threads and is prohibitively expensive to run on the *All of Us* dataset. **(b)** Detailed runtime breakdown of BOLT-LMM-inf runs on the *All of Us* dataset.

## Data Availability

The individual-level *All of Us* data are available to authorized researchers through the All of *Us* Researcher Workbench under its controlled tier access framework and cannot be redistributed by the authors; access is granted by the *All of Us* Research Program following registration and completion of its data use requirements. The 1000 Genomes data are publicly available [33]. The .vcf.gz files can be downloaded from https://ftp.1000genomes.ebi.ac.uk/vol1/ftp/data_collections/1000G_2504_high_coverage/working/20220422_3202_phased_SNV_INDEL_SV/. The simulated 200,000- and 500,000-individual cohorts can be downloaded from https://portal.nersc.gov/cfs/m4341/mikado/, which includes the GRG files and the PLINK files for the last four chromosomes of the 200k cohort. The analyses were conducted within the *All of Us* Researcher Workbench in a controlled-tier workspace (ID test-workspace-grg-gg-s-pod, UUID 8680c880-37a5-471c-b5e5-1b965ce196cd), provisioned in resource region us-central1 on pod user-pod-gguidi-0ef9.

## Footnotes

1 Mikado refers to the pick-up-sticks game, in which a stick may be lifted only when no other stick rests on it.

2
https://github.com/CornellHPC/grapp/tree/c49cc2e37c0561cbd336b248b4091bb96782f897

3 Details on GRG construction can be found at https://grgl.readthedocs.io/en/stable/construct.html.

## Code Availability

The GRG-related repositories necessary to reproduce the results from this paper are consolidated at https://github.com/CornellHPC/mikado/releases/tag/v0.2. This includes pinned versions of grgl, grapp, and grg-spmv, which are the three packages necessary to run Mikado and baseline grgl on the applications. Dockerfiles and benchmark scripts are also provided.
