# Sparse Linear Algebra Accelerates Genotype Representation Graph Computation at Biobank Scale

**DOI:** 10.64898/2026.09.10.750583

**Published:** 2026-10-31

**Authors:** Yifan Li, Qingyao Sun, Drew DeHaas, Max Xiaohang Zhao, Adam A. Boyko, Shaila A. Musharoff, Xinzhu Wei, Giulia Guidi

## Abstract

Biobank-scale genomic analyses are increasingly constrained by computational costs, as hundreds of thousands to millions of samples and variants must be analyzed together. The genotype representation graph (GRG) compactly encodes population genetic variation to accelerate computation, but its graph-traversal implementation does not exploit modern accelerators. This work introduces Mikado, a methodology that expresses GRG-based computation using sparse linear algebra primitives. Under a reverse topological ordering, the GRG adjacency matrix is strictly block-lower-triangular, and the genotype matrix-vector product becomes a sparse triangular solve, decomposed into a pipelined sequence of blocked sparse matrix-vector multiplies. Because GRGs are shallow by construction, the solve completes in only a few sequential stages, exposing fine-grained parallelism and enabling vendor-optimized sparse primitives on GPUs. On the full All of Us cohort, Mikado computes principal components in minutes, where PLINK2 cannot complete one chromosome in 72 hours, and runs genome-wide BOLT-LMM-inf in under 19 minutes on one GPU, where the official BOLT-LMM analysis would be impractical. Our two-stage reformulation is independent of the hardware: the same code runs unchanged across GPU architectures and, on CPUs, gives a 1.8x single-threaded speedup over the existing GRG library together with multi-threaded scaling, providing a scalable, portable approach for population genetics as biobanks continue to grow.

## Full Text Availability

The license terms selected by the author(s) for this preprint version do not permit archiving in PMC. The full text is available from the preprint server.

