# Supplementary material for: Sparse Linear Algebra Accelerates Genotype Representation Graph Computation at Biobank Scale
Source: bioRxiv. 2026 Sep 21:2026.09.10.750583. Preprint. [Version 2] doi: 10.64898/2026.09.10.750583 (PMC13622022; doi:10.64898/2026.09.10.750583)
Supplement: Supplementary file 1 [file NIHPP2026.09.10.750583V2-supplement-1.pdf]

bioRxiv preprint doi: https://doi.org/10.64898/2026.09.10.750583; this version posted September 21, 2026. The copyright holder for this preprint (which was not certified by peer review) is the author/funder, who has granted bioRxiv a license to display the preprint in perpetuity. It is made available under a CC-BY 4.0 International license.

Supplementary Information

Extended Data Fig. 1 | Kernel Performance on More Machines

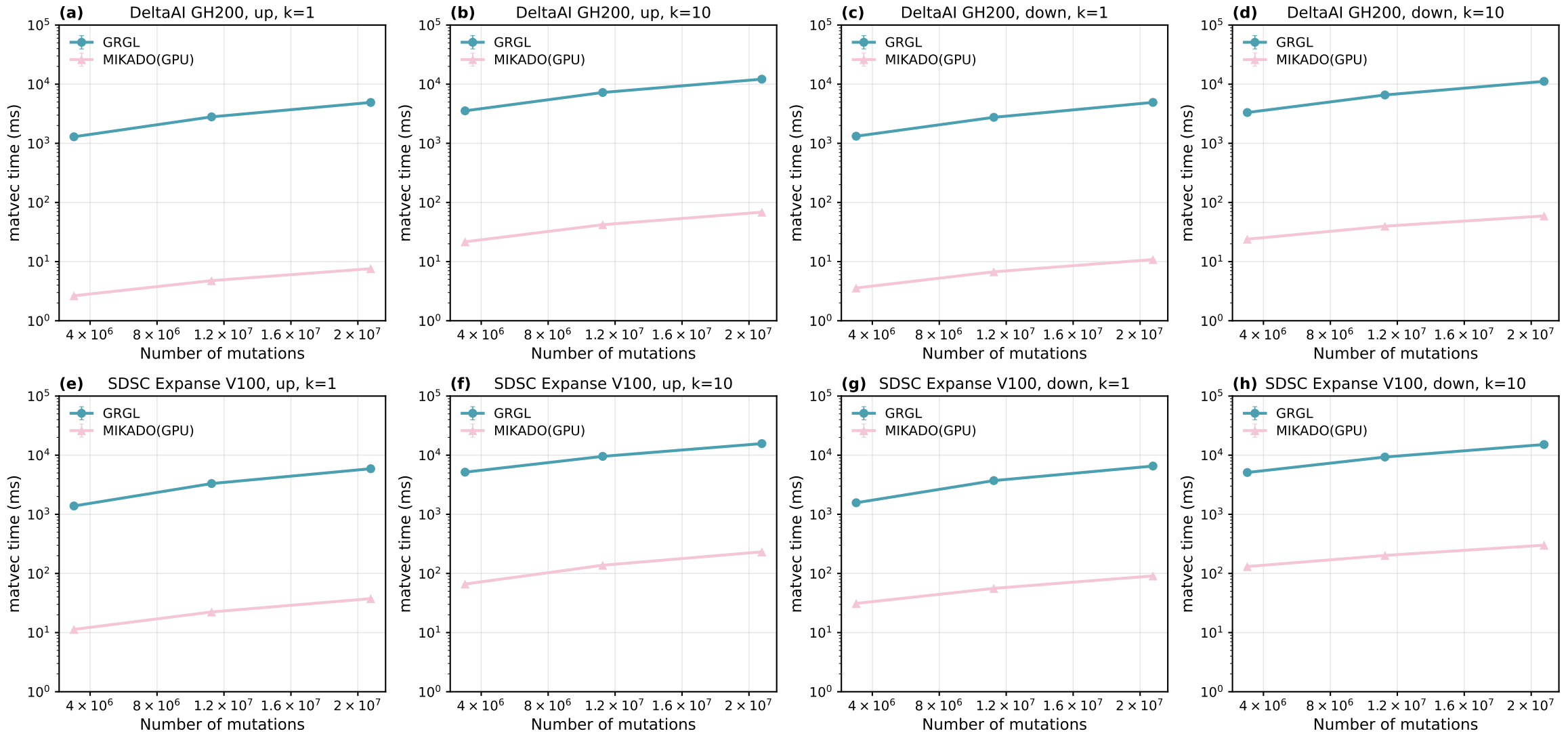

Extended Figure 1 MIKADO GPU Performance on DeltaAI and SDSC Exapnse The kernel benchmark is done on chromosome 1,11,21 of Simulated 500K dataset. These results show that MIKADO works on GPUs of different generations and has great portability **(a-d)** Performance on DeltaAI, with Arm Neoverse V2 CPU and H100 GPU. MIKADO GPU achieves an average speedup of 477× on $k = 1$ runs and an average speedup of 166× on $k = 10$ runs. **(e-h)** Performance on SDSC Expanse, with Intel 6248 Xeon V2 CPU and V100 GPU. MIKADO GPU achieves an average speedup of 93× on $k = 1$ runs and an average speedup of 56× on $k = 10$ runs.

Extended Data Fig. 2 | Streaming Backend Performance

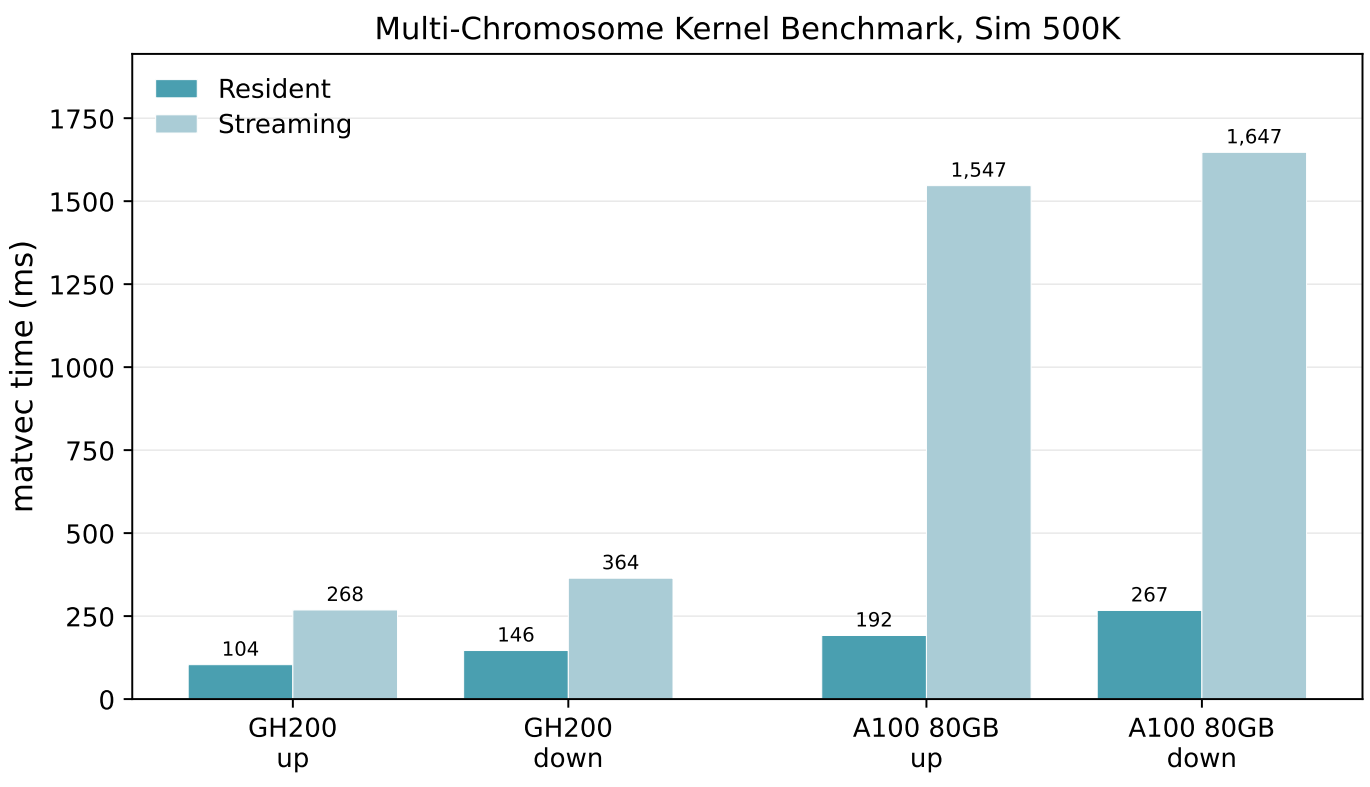

Extended Figure 2 Streaming Backend Comparison. By default, all GRGs reside in GPU memory; we refer to this as resident mode. For GRGs too large to fit in device memory, MIKADO streams blocks on demand from host memory with double buffering, so one block is processed while the next is in transit. We call this streaming mode. This significantly reduces GPU memory requirements; performance is bound by host-to-device bandwidth rather than compute in this mode. We perform multi-chromosome kernel benchmarks on DeltaAI (one GH200 superchip) and Perlmutter (one A100 80 GiB) with the Simulated 500K dataset, in both resident and forced-streaming mode to isolate the cost of streaming. On the A100, connected over PCIe 4.0 at 32 GB/s, forcing streaming incurs a slowdown exceeding 6×. On the GH200 superchip, which pairs a 72-core Grace CPU with an H100 GPU over an 450 GB/s NVLink-C2C interface, the slowdown falls below 3×.

bioRxiv preprint doi: https://doi.org/10.64898/2026.09.10.750583; this version posted September 21, 2026. The copyright holder for this preprint (which was not certified by peer review) is the author/funder, who has granted bioRxiv a license to display the preprint in perpetuity. It is made available under a CC-BY 4.0 International license.

Extended Data Fig. 3 | Genome-Wide Association Study (GWAS) Runtime and Cost

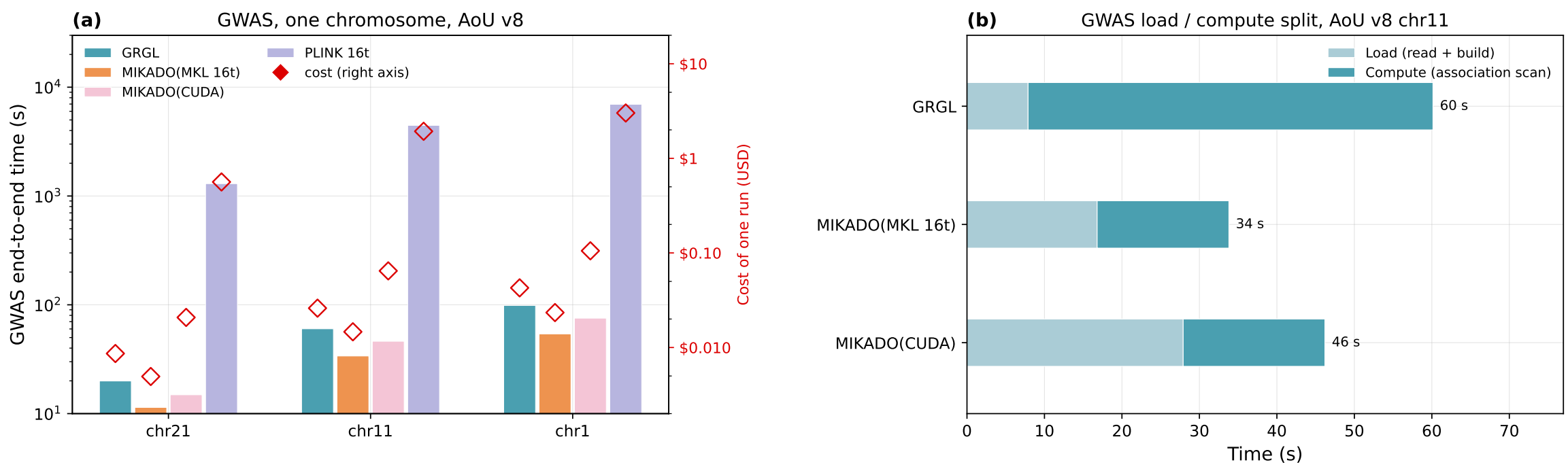

Extended Figure 3 Runtime and Cost of GWAS. GWAS tests each variant for association with a phenotype, conditional on covariates; for a quantitative phenotype, the core per-variant computation reduces to a single $G^{\top}w$ product followed by statistical post-processing. Runtime is therefore dominated by chromosome loading rather than by the matrix–vector kernel, placing GWAS at the low end of the workload intensity range described in the main text. **(a)** A single-chromosome GWAS runs on *All of Us* v8. On chromosome 11, Mikado CPU completes GWAS end to end in 34 s, compared with 4,457 s for PLINK2 and 60 s for the CPU GRG baseline, a 132× speedup over PLINK2 and 1.7× over the CPU GRG baseline; chromosomes 1 and 21 show the same pattern. Output write time is excluded for all implementations, and MKL's `optimize` is disabled. **(b)** Runtime breakdown for chromosome 11, where Mikado on CPU is faster than on GPU and achieves the lowest cost.

bioRxiv preprint doi: https://doi.org/10.64898/2026.09.10.750583; this version posted September 21, 2026. The copyright holder for this preprint (which was not certified by peer review) is the author/funder, who has granted bioRxiv a license to display the preprint in perpetuity. It is made available under a CC-BY 4.0 International license.

Extended Data Fig. 4 | Numerical Agreement with Official BOLT-LMM-inf

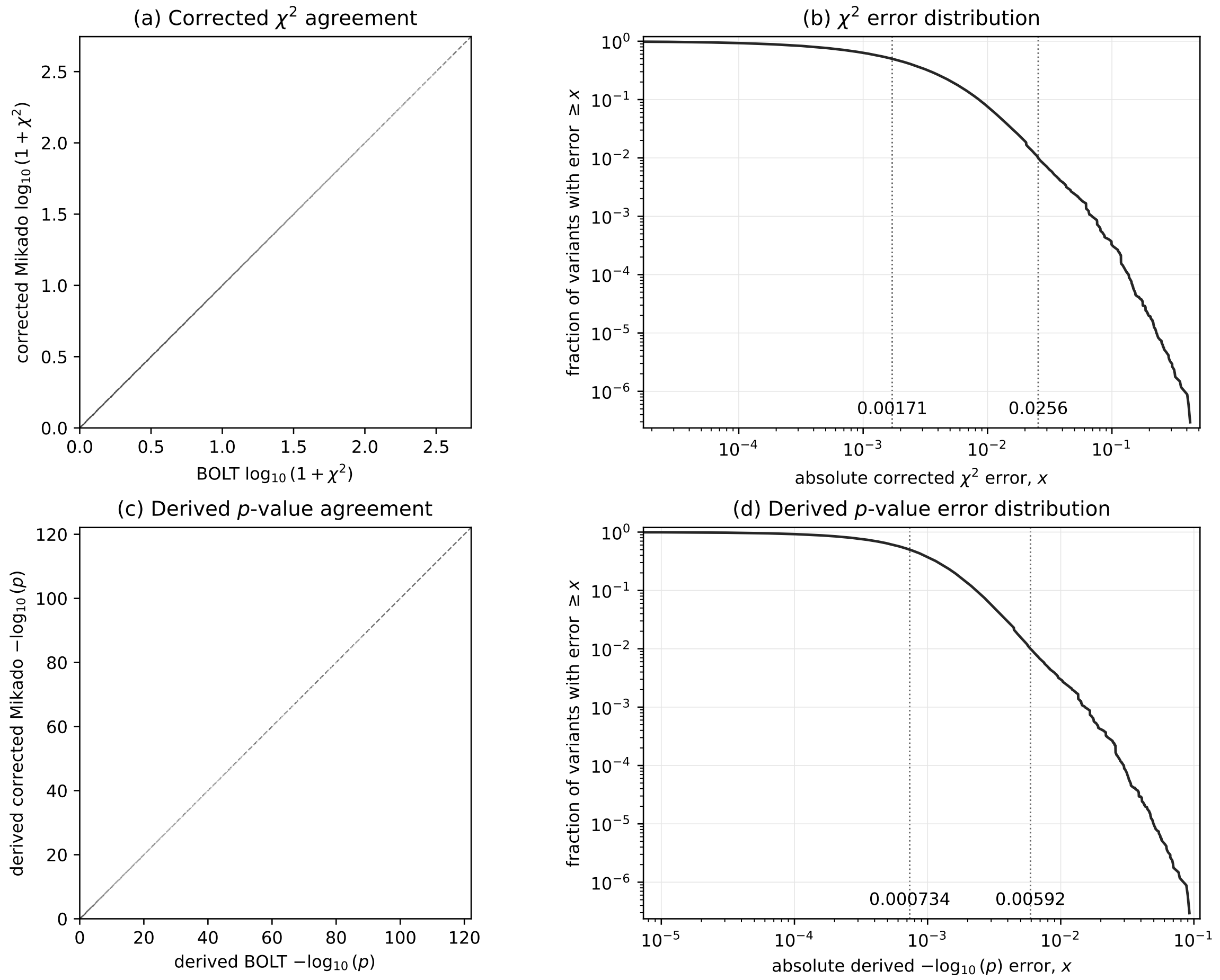

Extended Figure 4 The numerical correctness of BOLT-LMM association statistics. Comparison of official BOLT-LMM and Mikado GPU for the 200K simulation on chromosomes 19–22 after placing Mikado's statistics on BOLT's calibration scale. **(a)** The square-bin density of corrected χ^2 statistics; the dashed line is the identity. **(b)** The empirical exceedance distribution of the absolute χ^2 error. **(c,d)** The corresponding agreement and absolute error distributions for the derived p values. In both error panels **(b,d)**, the left and right dotted guides mark the median and 99th-percentile errors, respectively, with their values printed on the x-axis. Each output row tests one additive SNP coefficient, so its squared standardized score follows a χ_1^2 distribution. The p-values are recomputed from χ^2 using the same χ_1^2 survival function because BOLT's textual values are heavily rounded. For this diagnostic comparison only, Mikado statistics are placed on BOLT's realized calibration scale to isolate implementation-level agreement; this rescaling is not part of the Mikado workflow and is not required in practice.

Supplementary Note 5 | Numerical Analysis of BOLT-LMM

Our Mikado GPU implementation of BOLT-LMM differs from the official implementation in three respects: it uses a lossless GRG representation of the genotypes, algebraically equivalent kernels with a different floating-point reduction order, and NumPy PRNG rather than Boost PRNG to generate the Monte Carlo probes and calibration SNP samples. The representation is exact, and the altered reduction order affects only numerical rounding. The different PRNG changes the realized stochastic samples even when both programs receive the same seed, so two otherwise equivalent runs can produce slightly different heritability estimates and overall calibration factors.

Each of the 3,391,600 variants on chromosomes 19–22 produces outputs that match one-to-one. In the illustrated simulation with target $h^2 = 0.6$, BOLT and Mikado estimate $\widehat{h^2}$ as 0.579444 and 0.577319, respectively. Importantly, Mikado does not require a BOLT-LMM reference result or any additional rescaling in practice. The heritability-recovery experiment shows that its Monte Carlo estimation and calibration procedure accurately recovers the simulated heritability targets, establishing that the method independently produces useful inference.

bioRxiv preprint doi: https://doi.org/10.64898/2026.09.10.750583; this version posted September 21, 2026. The copyright holder for this preprint (which was not certified by peer review) is the author/funder, who has granted bioRxiv a license to display the preprint in perpetuity. It is made available under a CC-BY 4.0 International license.

Extended Data Fig. 4 addresses a separate implementation question: once the known, run-specific calibration-scale difference is factored out for this diagnostic comparison, do the accelerated representation and kernels reproduce BOLT's variant-level calculations? The independently sampled calibration factors are 1.09367 (BOLT) and 1.053465 (Mikado GPU). For this audit only, applying their ratio of 1.038164 places the statistics on a common realized calibration scale. The corrected statistics then agree essentially point for point, with a through-origin slope of 0.999955 and $R^2 = 0.999999871$. The absolute χ^2 errors have median, 99th percentile, and maximum values of 0.00171, 0.0256, and 0.425, respectively, and a relative L_2 error of 0.0356%. Derived p-values yield 45,818 shared genome-wide significant calls at 5×10^{-8} and only 17 boundary-crossing differences among 3.39 million tests.

The recovery experiment establishes that Mikado BOLT-LMM is valid without reference correction, while the pointwise comparison shows that the accelerated computation itself introduces a negligible additional numerical error. Together, these results show that the reported speedups do not compromise inferential accuracy.

bioRxiv preprint doi: https://doi.org/10.64898/2026.09.10.750583; this version posted September 21, 2026. The copyright holder for this preprint (which was not certified by peer review) is the author/funder, who has granted bioRxiv a license to display the preprint in perpetuity. It is made available under a CC-BY 4.0 International license.

Extended Section 1 | Per-Chromosome Statistics

In this section, we provide detailed per-chromosome statistics for each dataset in the form of tables.

Supplementary Table 1 AoU v8 phased dataset statistics. Cohort size: 414,830. PLINK2 is the total size of the `.pgen`/`.psam`/`.pvar` file set. PLINK2 is built only for a subset of chromosomes.

Chr	#Mutations	VCF.gz	PLINK2	GRG	GRG-SpMV	#Nodes	#Edges
1	69,165,511	238 GB	86 GB	8.3 GB	16 GB	178,677,711	2,026,690,586
2	76,695,484	259 GB		8.7 GB	16 GB	182,468,494	2,077,649,352
3	63,328,899	218 GB		7.1 GB	13 GB	150,127,703	1,692,271,935
4	61,362,449	216 GB		6.9 GB	13 GB	145,737,061	1,646,949,664
5	57,032,792	196 GB		6.4 GB	12 GB	136,152,008	1,536,836,594
6	53,288,431	191 GB		6.1 GB	12 GB	128,666,201	1,456,900,356
7	50,416,022	175 GB		6.1 GB	12 GB	128,581,218	1,488,117,888
8	49,585,609	167 GB		5.6 GB	11 GB	117,857,735	1,365,867,818
9	39,350,131	133 GB		5.0 GB	9.2 GB	107,003,860	1,278,132,610
10	42,213,334	149 GB		5.2 GB	9.6 GB	111,955,468	1,303,030,322
11	43,024,023	149 GB	55 GB	5.0 GB	9.2 GB	106,533,797	1,217,490,442
12	41,172,758	144 GB		5.0 GB	9.1 GB	106,381,765	1,211,686,472
13	30,469,204	108 GB		3.7 GB	6.8 GB	79,740,553	903,900,867
14	27,959,778	98 GB		3.5 GB	6.4 GB	74,262,590	866,463,284
15	25,724,763	89 GB		3.5 GB	6.5 GB	76,098,461	916,442,328
16	29,179,546	95 GB		3.8 GB	7.0 GB	82,276,293	974,608,659
17	24,574,646	84 GB		3.4 GB	6.3 GB	75,226,753	886,785,072
18	23,870,313	84 GB		3.1 GB	5.6 GB	67,546,265	772,505,926
19	18,727,782	66 GB		2.8 GB	5.1 GB	61,060,691	728,921,352
20	19,247,900	67 GB		2.6 GB	4.8 GB	57,416,369	669,478,407
21	11,974,519	41 GB	16 GB	1.8 GB	3.3 GB	37,155,828	487,056,362
22	11,973,519	42 GB		1.9 GB	3.5 GB	40,213,718	520,556,991
**Total**	870,337,413	2.94 TB		106 GB	197 GB	2,251,140,542	26,028,343,287

bioRxiv preprint doi: https://doi.org/10.64898/2026.09.10.750583; this version posted September 21, 2026. The copyright holder for this preprint (which was not certified by peer review) is the author/funder, who has granted bioRxiv a license to display the preprint in perpetuity. It is made available under a CC-BY 4.0 International license.

Supplementary Table 2 AoU v8 phased, filtered dataset statistics used for the PCA experiment (chr21 only). Cohort size: 414, 830. The multi-allelic and non-SNP sites are removed.

Chr	#Mutations	PLINK2	GRG	GRG-SpMV	#Nodes	#Edges
1	36,917,066	41 GB	6.7 GB	12 GB	161,731,460	1,717,867,968
11	22,395,732	25 GB	4.0 GB	7.2 GB	95,759,938	1,036,625,578
21	5,851,262	7.0 GB	1.4 GB	2.5 GB	32,446,275	387,518,340

Supplementary Table 3 AoU v8 phased filtered dataset II used for the BOLT-LMM experiment. Cohort size: 220, 760. The sites with MAF $\geq 5 \times 10^{-5}$ and Hardy–Weinberg equilibrium $p \geq 10^{-12}$ are retained; multi-allelic and non-SNP sites are removed; The individuals were restricted to the EUR group in the ancestry file, excluding those with quality-control flags.

Chr	#Mutations	GRG	GRG-SpMV	#Nodes	#Edges
1	3,619,944	2.0 GB	3.7 GB	39,735,582	708,528,044
2	3,913,024	2.0 GB	3.8 GB	40,835,120	732,770,801
3	3,226,487	1.7 GB	3.1 GB	34,118,223	601,630,061
4	3,121,749	1.7 GB	3.1 GB	33,165,115	592,890,256
5	2,937,757	1.5 GB	2.9 GB	31,012,135	551,858,873
6	2,792,088	1.5 GB	2.8 GB	29,473,889	526,576,298
7	2,593,774	1.5 GB	2.7 GB	28,840,247	524,975,255
8	2,428,240	1.3 GB	2.5 GB	25,456,569	474,583,211
9	1,943,053	1.2 GB	2.2 GB	22,420,483	431,122,970
10	2,206,388	1.3 GB	2.4 GB	24,887,211	456,271,903
11	2,194,556	1.2 GB	2.3 GB	24,041,465	433,277,839
12	2,141,418	1.2 GB	2.2 GB	23,866,859	431,163,245
13	1,586,417	883 MB	1.7 GB	17,800,704	321,096,982
14	1,456,131	803 MB	1.6 GB	15,924,201	297,762,981
15	1,313,544	776 MB	1.5 GB	15,174,935	294,099,974
16	1,362,787	830 MB	1.6 GB	16,333,812	313,652,422
17	1,311,961	793 MB	1.5 GB	15,707,133	296,683,801
18	1,254,076	737 MB	1.4 GB	14,802,780	270,155,968
19	973,237	631 MB	1.2 GB	12,382,695	239,630,388
20	1,026,488	620 MB	1.2 GB	12,340,651	229,143,277
21	603,268	385 MB	752 MB	7,175,678	151,465,316
22	620,556	413 MB	811 MB	7,583,591	164,855,247
**Total**	44,626,943	25 GB	47 GB	493,079,078	9,044,195,112

bioRxiv preprint doi: https://doi.org/10.64898/2026.09.10.750583; this version posted September 21, 2026. The copyright holder for this preprint (which was not certified by peer review) is the author/funder, who has granted bioRxiv a license to display the preprint in perpetuity. It is made available under a CC-BY 4.0 International license.

Supplementary Table 4 1000 Genomes dataset statistics. Cohort size: $3,202$. PLINK1 is the total size of the `.bed`/`.bim`/`.fam` fileset.

Chr	#Mutations	VCF.gz	PLINK1	GRG	GRG-SpMV	#Nodes	#Edges
1	5,759,060	2.3 GB	4.5 GB	382 MB	875 MB	9,789,561	95,697,894
2	6,088,598	2.4 GB	4.8 GB	392 MB	896 MB	9,874,575	95,938,111
3	4,983,185	2.0 GB	4.0 GB	320 MB	734 MB	8,019,712	78,820,046
4	4,875,465	2.0 GB	3.9 GB	308 MB	704 MB	7,705,185	73,945,788
5	4,536,816	1.8 GB	3.5 GB	290 MB	663 MB	7,283,724	70,678,135
6	4,315,215	1.8 GB	3.4 GB	277 MB	638 MB	6,939,935	68,945,517
7	4,137,254	1.7 GB	3.2 GB	276 MB	635 MB	7,044,360	70,184,271
8	3,886,222	1.6 GB	3.0 GB	249 MB	570 MB	6,214,910	61,227,189
9	3,165,513	1.3 GB	2.5 GB	217 MB	503 MB	5,669,765	56,925,342
10	3,495,473	1.4 GB	2.8 GB	235 MB	542 MB	6,008,320	60,438,238
11	3,423,341	1.4 GB	2.7 GB	221 MB	509 MB	5,591,167	55,020,194
12	3,332,788	1.4 GB	2.6 GB	223 MB	513 MB	5,728,239	56,634,359
13	2,509,179	1.0 GB	2.0 GB	166 MB	384 MB	4,290,223	42,002,909
14	2,290,397	922 MB	1.9 GB	154 MB	355 MB	3,991,217	39,356,334
15	2,109,283	845 MB	1.7 GB	150 MB	350 MB	3,995,081	40,578,358
16	2,362,361	917 MB	1.9 GB	165 MB	382 MB	4,352,277	43,666,085
17	2,073,624	823 MB	1.7 GB	150 MB	349 MB	4,049,440	40,601,862
18	1,963,845	796 MB	1.6 GB	134 MB	312 MB	3,505,457	35,174,107
19	1,670,691	685 MB	1.4 GB	124 MB	290 MB	3,367,667	34,275,773
20	1,644,383	656 MB	1.4 GB	116 MB	269 MB	3,094,018	30,844,353
21	1,002,752	408 MB	801 MB	74 MB	173 MB	2,002,216	20,626,977
22	1,066,556	426 MB	851 MB	80 MB	188 MB	2,201,197	22,596,237
**Total**	70,692,001	28 GB	56 GB	4.6 GB	11 GB	120,718,246	1,194,178,079

Supplementary Table 5 1000 Genomes filtered dataset statistics. Cohort size: $3,202$. The sites with MAF $\geq 10^{-2}$ are removed; multi-allelic and non-SNP sites are removed.

Chr	#Mutations	PLINK1	GRG	GRG-SpMV	#Nodes	#Edges
1	965,732	769 MB	210 MB	446 MB	7,065,206	63,333,825
2	1,017,890	811 MB	215 MB	458 MB	7,117,446	65,117,678
3	866,798	691 MB	177 MB	379 MB	5,812,305	54,086,466
4	878,077	699 MB	170 MB	362 MB	5,580,506	50,342,902
5	782,046	623 MB	159 MB	340 MB	5,250,210	48,289,962
6	794,207	632 MB	155 MB	335 MB	5,053,659	47,972,464
7	723,384	576 MB	156 MB	335 MB	5,138,560	48,626,690
8	675,084	537 MB	137 MB	296 MB	4,485,794	42,468,110
9	548,407	436 MB	126 MB	273 MB	4,196,446	40,122,484
10	623,090	497 MB	133 MB	289 MB	4,390,983	41,924,084
11	603,792	482 MB	124 MB	268 MB	4,067,642	38,477,651
12	586,049	468 MB	126 MB	270 MB	4,195,510	38,799,836
13	444,065	355 MB	93 MB	202 MB	3,120,407	28,924,435
14	399,126	319 MB	87 MB	189 MB	2,917,811	27,248,287
15	366,296	292 MB	89 MB	194 MB	2,986,710	28,813,222
16	382,381	306 MB	95 MB	206 MB	3,205,288	30,715,669
17	335,828	268 MB	87 MB	188 MB	2,994,097	27,950,002
18	347,752	278 MB	78 MB	169 MB	2,581,796	24,786,165
19	281,230	224 MB	73 MB	157 MB	2,490,249	23,294,882
20	282,329	225 MB	68 MB	146 MB	2,284,992	21,533,015
21	179,024	143 MB	45 MB	98 MB	1,488,689	14,785,617
22	183,023	146 MB	49 MB	106 MB	1,646,259	16,036,285
**Total**	12,265,610	9.5 GB	2.6 GB	5.6 GB	88,070,565	823,649,731

bioRxiv preprint doi: https://doi.org/10.64898/2026.09.10.750583; this version posted September 21, 2026. The copyright holder for this preprint (which was not certified by peer review) is the author/funder, who has granted bioRxiv a license to display the preprint in perpetuity. It is made available under a CC-BY 4.0 International license.

Supplementary Table 6 Simulated 200k dataset statistics. Cohort size: 200,000.

Chr	#Mutations	GRG	GRG-SpMV	#Nodes	#Edges
1	17,083,232	1.5 GB	2.9 GB	32,951,213	309,717,578
2	16,637,528	1.4 GB	2.8 GB	31,484,527	284,487,370
3	13,625,360	1.2 GB	2.3 GB	26,039,836	243,055,123
4	13,058,115	1.1 GB	2.2 GB	25,327,665	236,364,913
5	12,474,779	1.1 GB	2.1 GB	23,971,215	225,747,613
6	11,730,038	1011 MB	2.0 GB	22,587,449	213,923,794
7	10,960,584	970 MB	1.9 GB	21,690,881	210,715,273
8	9,953,740	882 MB	1.8 GB	19,561,095	193,397,364
9	9,500,000	847 MB	1.7 GB	18,533,938	191,235,205
10	9,196,528	869 MB	1.7 GB	19,537,600	198,856,189
11	9,265,101	820 MB	1.6 GB	18,130,386	180,033,855
12	9,158,771	856 MB	1.7 GB	19,173,025	195,939,104
13	6,598,679	630 MB	1.3 GB	13,834,971	148,396,050
14	6,034,574	596 MB	1.2 GB	12,972,483	145,908,504
15	5,656,206	629 MB	1.3 GB	13,865,922	167,617,037
16	6,224,437	629 MB	1.3 GB	13,628,263	159,058,378
17	5,714,000	601 MB	1.2 GB	13,119,720	154,601,782
18	5,523,349	559 MB	1.1 GB	12,139,949	140,091,898
19	4,010,942	479 MB	964 MB	10,311,874	135,031,919
20	4,415,250	486 MB	969 MB	10,549,213	129,168,016
21	2,502,559	306 MB	616 MB	6,088,637	91,467,812
22	2,441,331	332 MB	676 MB	6,696,671	104,365,793
**Total**	191,765,103	18 GB	35 GB	392,196,533	4,059,180,570

Supplementary Table 7 Simulated 200k filtered dataset statistics. Cohort size: 200,000. Sites with MAF $\geq 10^{-4}$ are removed.

Chr	#Mutations	PLINK1	GRG	GRG-SpMV	#Nodes	#Edges
19	1,016,718	48 GB	377 MB	771 MB	9,153,249	130,648,570
20	1,117,819	53 GB	376 MB	759 MB	9,314,525	124,934,949
21	638,234	30 GB	242 MB	496 MB	5,289,198	89,054,606
22	618,829	29 GB	268 MB	556 MB	5,912,762	101,491,930

Supplementary Table 8 Simulated 500k dataset statistics. Cohort size: 500,000.

Chr	#Mutations	GRG	GRG-SpMV	#Nodes	#Edges
1	20,769,289	2.1 GB	4.0 GB	46,026,894	468,916,211
2	20,205,851	2.0 GB	3.8 GB	43,658,912	427,330,611
3	16,541,902	1.7 GB	3.2 GB	36,318,030	375,881,430
4	15,865,658	1.6 GB	3.1 GB	35,148,353	368,334,094
5	15,148,360	1.5 GB	2.9 GB	33,253,490	350,317,217
6	14,248,401	1.5 GB	2.8 GB	31,425,705	334,271,994
7	13,296,591	1.4 GB	2.7 GB	30,071,822	329,546,973
8	12,106,619	1.3 GB	2.5 GB	27,077,962	304,925,641
9	11,593,486	1.3 GB	2.4 GB	26,007,829	306,957,558
10	11,173,189	1.3 GB	2.4 GB	27,089,894	313,091,390
11	11,259,696	1.2 GB	2.3 GB	25,315,430	290,486,081
12	11,127,551	1.3 GB	2.4 GB	26,633,545	309,080,942
13	8,009,106	937 MB	1.8 GB	19,177,193	246,468,052
14	7,325,817	900 MB	1.8 GB	18,092,477	248,323,688
15	6,847,885	952 MB	1.9 GB	19,241,764	281,814,674
16	7,541,581	941 MB	1.9 GB	19,038,920	262,937,910
17	6,947,200	914 MB	1.8 GB	18,340,394	263,842,135
18	6,709,681	857 MB	1.7 GB	17,074,882	242,590,328
19	4,870,210	745 MB	1.5 GB	14,367,819	234,885,598
20	5,371,399	760 MB	1.5 GB	14,815,210	228,835,097
21	3,034,975	519 MB	1.1 GB	8,673,158	180,180,643
22	2,967,369	557 MB	1.2 GB	9,483,299	199,325,274
**Total**	232,961,816	26 GB	51 GB	546,332,982	6,568,343,541

bioRxiv preprint doi: https://doi.org/10.64898/2026.09.10.750583; this version posted September 21, 2026. The copyright holder for this preprint (which was not certified by peer review) is the author/funder, who has granted bioRxiv a license to display the preprint in perpetuity. It is made available under a CC-BY 4.0 International license.

Extended Section 2 | Mikado Artifacts

The artifacts to reproduce the results in this paper can be downloaded from https://github.com/CornellHPC/Mikado/releases/tag/v0.2. This repository contains:

1. A README file.
2. Pinned version of GRGL (Genotype Representation Graph Library), which provides core functionalities such as constructing GRGs, as well as the baseline CPU backend.
3. Pinned version of `grapp`, which provides application implementations based on GRG.
4. Pinned version of GRG-SpMV, which provides the core functionalities of the Mikado backend for `grapp`.
5. `docker/` directory containing the dockerfiles for building the images.
6. `benchmark/` directory containing the benchmark scripts and config files.
7. `code/` directory containing auxiliary code files for evaluation purposes.

Docker-based setup is recommended, though manual installation is also possible. Here we provide examples for setting up and running with `podman-hpc` on the Perlmutter system; commands using `docker` are similar.

```
# Building the image
# Make sure you have cloned or downloaded the repo first
cd mikado/docker/mikado
podman-hpc build --no-cache -t mikado:latest .

# Saving the built image
podman-hpc save mikado:latest -o mikado.tar

# Loading the built image
podman-hpc load -i mikado.tar

# Converting chr11 from grg to grg_spmv format required for \mikado
# Links to download GRG files are provided in "Data Availability" section
podman-hpc run --rm \
    -v /PATH/TO/DATASET:/dataset \
    mikado:latest \
    python -c "
from pygrgl_spmv import simple_convert
simple_convert('/dataset/chr11.grg', '/dataset/chr11.grg_spmv')
    "

# Running benchmark with the provided benchmark scripts
# (Kernel benchmark, chr11, k=1, up, \mikado GPU backend, 1 GPU)
podman-hpc run --rm --gpu \
    -v /PATH/TO/DATASET:/dataset \
    -v /PATH/TO/RECORDS:/records \
    mikado:latest \
    bash -c '
        cd /opt/mikado/benchmark
        python3 evaluate.py -a kernel -b cusparse -d /dataset/chr11.grg_spmv \
            --warmup 1 --runs 1 \
            --record /records/kernel.jsonl \
            --device-map /opt/mikado/benchmark/configs/device_map_1.json \
            -- --direction up --k 1
    '
```

The README contains detailed instructions on how to install manually, build GRG, convert GRG, and run experiments with the benchmark scripts. The simulated datasets in `.grg` format can be downloaded from https://portal.nersc.gov/cfs/m4341/mikado/. If you need to build GRGs from `vcf.gz` format datasets, please refer to the GRGL documentation https://grgl.readthedocs.io/en/stable/construct.html.

bioRxiv preprint doi: https://doi.org/10.64898/2026.09.10.750583; this version posted September 21, 2026. The copyright holder for this preprint (which was not certified by peer review) is the author/funder, who has granted bioRxiv a license to display the preprint in perpetuity. It is made available under a CC-BY 4.0 International license.

Extended Section 3 | Parameters

Each backend in the benchmark suite may require specific parameters that affect performance and, in rare cases, precision. This section describes these parameter settings in detail. The main evaluation script (`evaluate.py`) is called with these parameters.

Mikado CPU (MKL):

1. `--mkl-threads <N>`: number of MKL threads per chromosome.
2. `--optimize`: enable MKL optimization flags.

The settings for these options are shown in Supplementary Table 9.

Supplementary Table 9 Mikado CPU (MKL) parameters.

Application	Reference Figure	`--mkl-threads`	`--optimize`
Kernel	Fig. 2 (a–f)	1	Yes
Kernel	Fig. 2 (g–h)	Varies	Varies
PCA	Fig. 3 (a,c)	16	Yes
PCA	Fig. 3 (b)	1	No
PCA	Fig. 3 (d)	1	Varies
BOLT-LMM	Fig. 4	1	No
GWAS	Extended Fig. 3	16	No

Mikado GPU (cuSparse):

1. `--native`: Use CuPy arrays, GPU-to-GPU communication, and CuPy-based PCA; set for all experiments except GWAS (Extended Fig. 3).
2. `--capture`: Enable CUDA graph capture; set for all experiments.
3. `--device-map <FILE>`: Control the mapping of chromosomes onto devices, used to control the number of GPUs in benchmarks. `device_map_1.json` is used for all single-chromosome experiments.
4. `--force-spmm`: Forces $k = 2$ when computing $k = 1$ to avoid a cuSparse precision bug for SpMV in earlier CUDA versions; **not** set for any experiment.
5. `--tol-record <FILE>`: A record file used to align stopping criteria for CuPy-based PCA runs; set to the corresponding record file from GRGL runs for PCA experiments.
6. `--no-allow-residency --vram-budget-mb <N>`: Used together to set a GPU memory budget and force streaming mode; set only for the Streaming Backend Comparison (Extended Fig. 2), for which the GPU memory budget is 8000 MB.

The settings for the device-map option in multi-chromosome experiments are shown in Supplementary Table 10.

Supplementary Table 10 Mikado GPU (cuSparse) parameters.

Application	Figure	`--device-map (device_map_{n}.json)`
PCA	Fig. 3 (b)	4
BOLT-LMM	Fig. 4	1

PLINK2:

1. `--threads <N>`: The number of threads.

The settings for the thread option are shown in Supplementary Table 11. Below are the commands the benchmark scripts use to invoke PLINK2.

Invoke command for PLINK2 PCA:

```
plink2 \
    --bfile <MERGED_FILE> \
    --pca <N> approx \
    --threads 32 \
    --out <FILE>
```

bioRxiv preprint doi: https://doi.org/10.64898/2026.09.10.750583; this version posted September 21, 2026. The copyright holder for this preprint (which was not certified by peer review) is the author/funder, who has granted bioRxiv a license to display the preprint in perpetuity. It is made available under a CC-BY 4.0 International license.

Invoke command for PLINK2 GWAS:

```
plink2 \
    --bfile <FILE> \
    --glm allow-no-covars hide-covar \
    --pheno <FILE> \
    --threads <N> \
    --out <FILE>
```

Supplementary Table 11 **PLINK2** parameters.

Application	Figure	`--threads`
PCA	Fig. 3 (a)	16
PCA	Fig. 3 (b)	64
GWAS	Extended Fig. 3 (a)	16

BOLT-LMM:

1. `--threads <N>`: The number of threads.

The settings for the thread option are shown in Supplementary Table 12. Below are the commands the benchmark scripts use to invoke BOLT-LMM.

```
bolt \
    --fam <FILE> \
    --phenoFile <FILE> \
    --phenoCol <S> \
    --lmmInfOnly \
    --verboseStats \
    --statsFile <FILE> \
    --numThreads <N> \
    --seed 2026 \
    --noMapCheck \
    --maxModelSnps <N_TOTAL_SNPS> \
    --bim <FILE> \
    ...
    --bed <FILE> \
    ...
```

Supplementary Table 12 **BOLT-LMM** parameters.

Application	Figure	`--threads`
BOLT-LMM	Fig. 4	64

bioRxiv preprint doi: https://doi.org/10.64898/2026.09.10.750583; this version posted September 21, 2026. The copyright holder for this preprint (which was not certified by peer review) is the author/funder, who has granted bioRxiv a license to display the preprint in perpetuity. It is made available under a CC-BY 4.0 International license.

Extended Section 4 | GPU Memory Consumption and Requirement

Mikado GPU does not introduce significant additional memory overhead compared to the CPU version. GPU memory consumption comprises three components: the GRG, the input and output vectors, and the working memory.

1. The GRG occupies approximately the same space in GPU memory as it does on disk, plus the calculation buffer at each node;
2. The total size of the input and output vectors depends on the block width k and the data type;
3. The working memory can be classified into setup memory (for CUDA graphs, aliased value buffers, etc.) and application-specific memory.

The working memory remains well below the GRG size for the applications we evaluated. In Table 13, we provide the calculated sizes of (1) and (2), along with the measured maximum GPU memory usage across three different experiments.

Supplementary Table 13 GPU memory consumption.

Application	Dataset	GRG Size	I/O Size	Total GRG + I/O	Measured Max.
GWAS	Sim 500k (Chr. 1)	4.3 GB	0.2 GB	4.5 GB	5.2 GB
PCA	1000 Genomes	11.9 GB	0.5 GB	12.4 GB	13.3 GB
BOLT-LMM	1000 Genomes Filtered	6.3 GB	0.8 GB	7.1 GB	9.0GB

Extended Section 5 | Platform Configuration

In Table 14, we list the hardware configurations of the machines used in this work.

bioRxiv preprint doi: https://doi.org/10.64898/2026.09.10.750583; this version posted September 21, 2026. The copyright holder for this preprint (which was not certified by peer review) is the author/funder, who has granted bioRxiv a license to display the preprint in perpetuity. It is made available under a CC-BY 4.0 International license.

Supplementary Table 14 Platform configurations used in the experiments. V100 GPUs are connected by second-generation NVLink. A100 GPUs are connected by third-generation NVLink. GH200 nodes feature NVLINK-C2C. Cloud prices are based on rates at the time of writing.

Platform	Experiment type	Node type	CPU	Memory	GPUs	USD/h
All of Us Researcher Workbench (GCP)	Single-chromosome, CPU	`n2-standard-32`	32 vCPU / 16 phys. cores	128 GB	—	1.56
	Multi-chromosome, CPU	`n2-highmem-48`	48 vCPU / 24 phys. cores	384 GB	—	3.15
	GPU	GPU A100 80GB	12 vCPU / 6 phys. cores per GPU	170 GB per GPU	1×/4× A100 80 GiB SXM	5.03 / 20.12
Perlmutter	—	GPU node	AMD EPYC 7763, 64 cores	256 GB	4× A100 40 GB SXM†	—
DeltaAI	—	GH200 node	Grace ARM CPU, 72 cores	120 GB	1× H100 96 GB	—
SDSC Expanse	—	V100 node	2× Intel Xeon 6248, 40 cores in total	384 GB	4× V100 32 GB SXM2	—

† Except for the streaming backend comparison, (Extened Fig. 2) where A100 80 GB SXM is used.
